# Neuro-Mechanics of Recumbent Leg Cycling in Post-Acute Stroke Patients

**DOI:** 10.1007/s10439-016-1660-0

**Published:** 2016-06-01

**Authors:** Emilia Ambrosini, Cristiano De Marchis, Alessandra Pedrocchi, Giancarlo Ferrigno, Marco Monticone, Maurizio Schmid, Tommaso D’Alessio, Silvia Conforto, Simona Ferrante

**Affiliations:** 1 Neuroengineering and Medical Robotics Laboratory, Department of Electronics, Information and Bioengineering, Politecnico di Milano, Piazza Leonardo da Vinci 32, 20133 Milan, Italy; 2 Physical Medicine and Rehabilitation Unit, Scientific Institute of Lissone, Salvatore Maugeri Foundation, Institute of Care and Research (IRCCS), Lissone, Italy; 3 BioLab3, Department of Engineering, University Roma TRE, Rome, Italy; 4 Department of Public Health, Clinical and Molecular Medicine, University of Cagliari, Cagliari, Italy

**Keywords:** Electromyography, Muscle synergies, Motor control, Biomechanics, Pedaling, Hemiparesis

## Abstract

Cycling training is strongly applied in post-stroke rehabilitation, but how its modular control is altered soon after stroke has been not analyzed yet. EMG signals from 9 leg muscles and pedal forces were measured bilaterally during recumbent pedaling in 16 post-acute stroke patients and 12 age-matched healthy controls. Patients were asked to walk over a GaitRite mat and standard gait parameters were computed. Four muscle synergies were extracted through nonnegative matrix factorization in healthy subjects and patients unaffected legs. Two to four synergies were identified in the affected sides and the number of synergies significantly correlated with the Motricity Index (Spearman’s coefficient = 0.521). The reduced coordination complexity resulted in a reduced biomechanical performance, with the two-module sub-group showing the lowest work production and mechanical effectiveness in the affected side. These patients also exhibited locomotor impairments (reduced gait speed, asymmetrical stance time, prolonged double support time). Significant correlations were found between cycling-based metrics and gait parameters, suggesting that neuro-mechanical quantities of pedaling can inform on walking dysfunctions. Our findings support the use of pedaling as a rehabilitation method and an assessment tool after stroke, mainly in the early phase, when patients can be unable to perform a safe and active gait training.

**Electronic supplementary material:**

The online version of this article (doi:10.1007/s10439-016-1660-0) contains supplementary material, which is available to authorized users.

## Introduction

Cycling leg exercise has been strongly applied as a motor function and/or aerobic training method for stroke patients, as well as an assessment tool.^3^ The recovery of walking ability is the ultimate goal of post-stroke lower limb rehabilitation,^1^ but mainly in the early phase, patients might encounter difficulties in performing standard gait training because of the unilateral weakness, which prevents a safe postural upright control. The pedaling paradigm has several advantages: it minimizes postural control, is characterized by a constrained kinematic trajectory, requires less effort for training supervision, and shares a similar muscle activation pattern with walking.^23^

Cycling training improves lower limb motor abilities and strength,^17^,^21^ balance,^17^ and cardiovascular fitness^33^ after stroke. Some studies have also observed promising improvements in terms of walking endurance and gait speed.^19^,^33^ Different therapies, such as Functional Electrical Stimulation 1,5 and biofeedback,^14^,^22^ used together with cycling training, seem to enhance its therapeutic effects, but further investigations are needed to identify the task characteristics, which maximize locomotor improvements.^3^

Pedaling-based metrics have been defined to support motor recovery assessment during and at the end of a rehabilitation program.^2^,^9^,^10^,^18^,^22^ Cycling motor performance was evaluated in terms of mechanical work:^2^,^9^,^18^ stroke patients exhibited a reduced amount of work on the affected side due to the reduced positive work done during the down-stroke phase of the cycle and an exaggerated amount of negative work done during the up-stroke phase.^18^ An index of unbalance, which compares the work done by the two legs, was proposed to quantify the asymmetrical motion typical of hemiparetic patients.^2^ Other authors observed a reduction of cycling smoothness in stroke patients compared to healthy elderly subjects.^10^,^22^ Besides mechanical measurements, neuromuscular correlates of cycling performance have been studied through EMG analysis. Two distinct types of abnormalities correlated with the reduced work production were found: a prolonged excitation in the vastus medialis and a phase-advanced excitation in the rectus femoris and semimembranosus muscles.^18^ Asymmetrical muscle patterns between the two sides were also observed for both the rectus femoris^2^,^10^,^22^ and the biceps femoris muscle.^2^,^22^

Recently, decomposition techniques have been applied on EMG signals to identify motor patterns of co-activation among group of muscles, called motor modules or muscle synergies. Each synergy is represented by a spatial component, reflecting the composition of muscle co-activation, and a temporal component, revealing the module recruitment along movement execution. This analysis, successfully applied to different motor behaviors, supports the hypothesis of a low-dimensional modular organization at the central nervous system (CNS) level.^6^

Motor modules have been proposed as a means for assessing motor impairment and providing rational targets for novel rehabilitation strategies able to enhance cortical plasticity.^31^,^35^ After stroke, the number of motor modules on the affected side during walking is reduced and is negatively correlated with the level of motor impairment.^8^,^11^ The spatial composition of the fewer independent modules seems to derive from a merging (i.e., a simultaneous recruitment) of the healthy motor modules.^11^ This supports the hypothesis that the decrease of independence in the neuromuscular control is due to a disruption in the descending neural commands.^35^

The muscle coordination adopted by healthy subjects during upright cycling has been explored in different studies. The coordination of leg muscles of professional cyclists was well represented by the activation of three modules, which were robust across subjects, power outputs, and pedaling positions.^16^ A similar modular coordination was identified in untrained subjects, during both upright^12^,^13^ and recumbent pedaling.^4^ These studies, as well as others dealing with different motor tasks, such as perturbed posture^30^ or mechanically altered walking,^24^ revealed how the analysis of motor modules in conjunction with mechanical measurements can provide an integrated insight onto the neural strategies adopted by the CNS for the accomplishment of a specific task.

Despite the aforementioned importance of cycling in post-stroke rehabilitation,^3^ how the modular control of cycling is altered after stroke has not been analyzed yet. The first aim of this study was to investigate whether stroke patients and age-matched healthy controls exhibited a modular muscle coordination during recumbent pedaling. Specifically, we predicted that, soon after stroke, the unaffected side shared a common modular organization with age-matched healthy controls, while the affected side showed a reduction of the number of modules, negatively correlated with the level of motor impairment. The second aim was to demonstrate that the spatial composition was preserved soon after stroke for both sides, and that the reduction in the number of modules in the affected side was due to an altered simultaneous temporal recruitment of the healthy modules. Finally, we proposed a set of cycling-based neuro-mechanical metrics able to provide a deeper quantitative analysis of lower limb motor impairment after stroke and we evaluated whether these metrics were representative of specific walking dysfunctions.

## Materials and Methods

### Participants

Twelve healthy subjects with an age of >60 years and no previous history of neurological injury, and 16 post-acute stroke patients were recruited. Inclusion criteria for patients were: adult age; first unilateral stroke, either ischemic or hemorrhagic, occurred <6 months before recruitment. Patients were excluded in case of major cognitive deterioration (Mini Mental State Examination score <24), cardiovascular and/or respiratory dysfunctions contraindicative of pedaling (e.g., recent ischemic heart attack, uncontrolled arterial hypertension, oxygen saturation of <90%), systemic diseases, and neurodegenerative or neuromuscular diseases.

The study was approved by the hospital’s Institutional Review Board and conducted in conformity with ethical and human principles of research. All subjects gave their written consent to participate.

#### Clinical Scales and Gait Parameters

Lower limb motor impairment was evaluated through the leg subscale of the Motricity Index (MI), which evaluates the strength of the affected leg and ranges from 0 (maximal impairment) to 100 (no impairment). Patients were also involved in a walking test: they were asked to walk three times over the GaitRite mat (CIR Systems Inc.), an electronic walkway incorporating pressure-sensitive pads, at self-selected speed using their usual walking aid (if any) or receiving the required assistance. Spatio-temporal gait parameters (gait speed, stance time, and double support time) were measured through the GaitRite software. A gait symmetry index (ST ratio) was computed as the ratio between the percentage stance time of the affected and unaffected leg.^14^

### Experimental Setup

**Figure 1:**
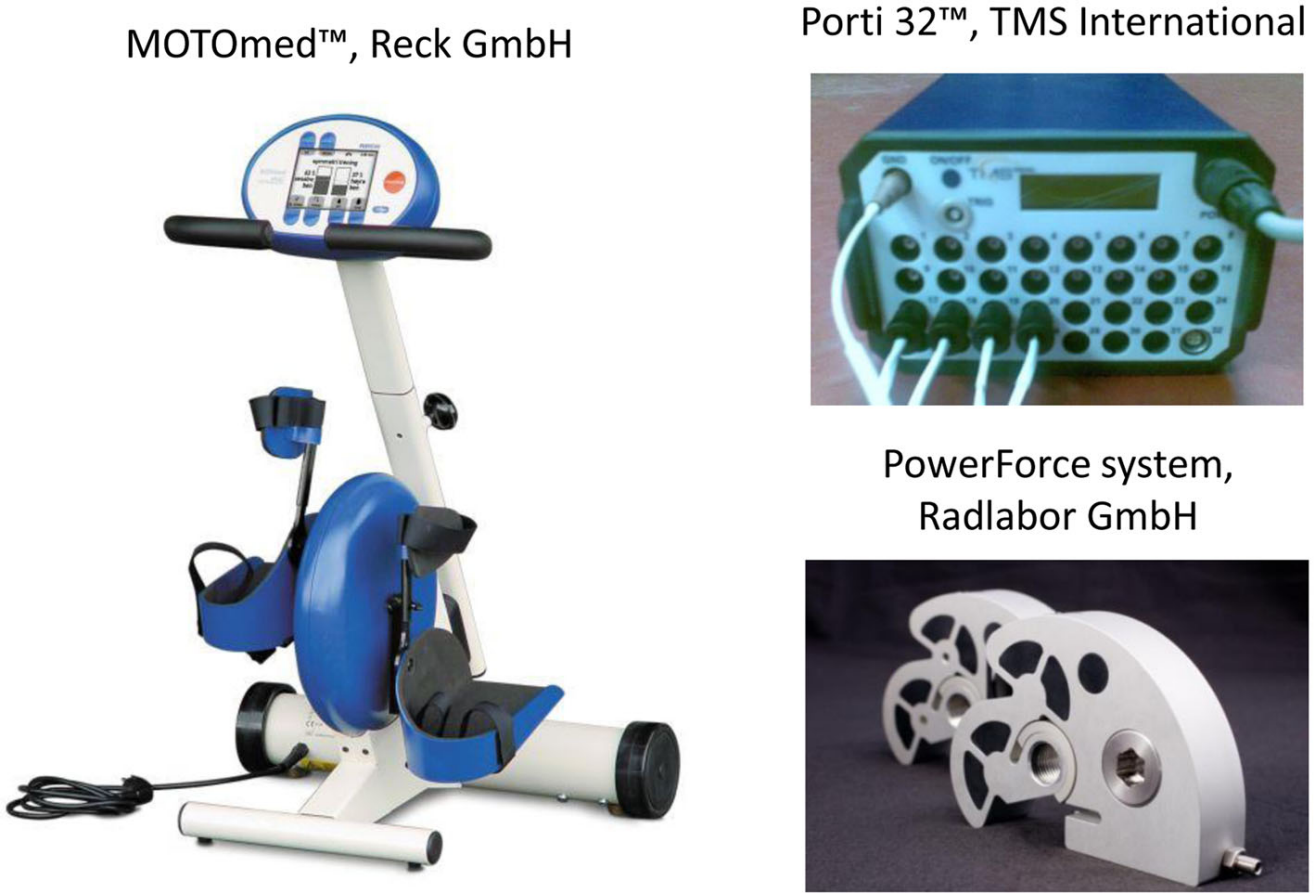
Experimental setup.

The experimental setup included a recumbent motorized cycle-ergometer (MOTOmed™, Reck GmbH) and two devices (PowerForce system, Radlabor GmbH), mounted between the crank and each pedal, to measure the force produced by each leg independently (Fig. 1). Each measuring device was based on a two-sensor system which measured the magnetic field variations (Hall effect) as a result of the displacement of a small sensor from a magnet. The sensor displacement was determined by the force applied at the pedal.^32^ At each pedal, since the two sensors were located orthogonally, both the tangential and the radial component of the force were measured. The same device was also equipped with accelerometers to measure the crank angle. Both the forces and the angle were sampled at 1000 Hz and synchronized with the EMG data through a digital output.

By using self-adhesive Ag/AgCl electrodes (Kendall™, COVIDIEN), surface EMG signals were recorded bilaterally from 9 leg muscles: Gluteus Maximus (Gmax), Biceps Femoris long head (BFlh), Biceps Femoris short head (BFsh), Gastrocnemius Medialis (GAS), Soleus (SOL), Tensor Fasciae Latae (TFL), Rectus Femoris (RF), Vastus Lateralis (VL), and Tibialis Anterior (TA). The skin was cleaned and the hair shaved to assure good contact; the electrodes were placed following SENIAM indications by the same researcher for all participants. A goniometer (Biometrics Ltd.) was used to measure the right knee flexion–extension angle. Both EMG signals and knee angle were acquired by a multi-channel signal amplifier (Porti 32™, TMS International) and sampled at 1024 Hz.

### Experimental Protocol

Subjects were seated on a chair in front of the ergometer and the distance was standardized to assure a maximum knee angle throughout the pedaling cycle of about 140°–150°. Each subject conducted four trials at different target cadences (20, 30, 40, and 50 RPM); the trials were separated by 5 min of rest to prevent fatigue and the execution order was randomized. Each trial lasted 3 min: 1-min of passive pedaling, during which the ergometer motor provided assistance at the target cadence and the subject was instructed not to voluntarily intervene in the movement, and 2-min of assisted voluntary pedaling, during which the motor provided assistance at 10 RPM less than the target cadence and the subject were encouraged to pedal with both legs and maintain the target pace. A visual numerical indicator and a metronome helped the subjects to keep the steady cadence.

### Data Analysis

Thirty pedaling cycles during the active phase with a cadence within ±4 RPM with respect to the target pace were selected. A pedaling cycle was defined as a complete revolution of the right crank and the zero angle corresponded to the right crank placed horizontally and backward. EMG signals were band-pass filtered (20–400 Hz, 3rd order Butterworth), full-wave rectified, and low-pass filtered at 5 Hz to calculate the envelope.^4^,^11^,^16^ Tangential and radial force signals were low-passed filtered at 10 Hz (3rd order Butterworth).

#### Muscle Synergies Extraction

The EMG envelopes of each cycle were expressed as a function of the crank angle and re-sampled on a 360-point vector by means of a cubic spline approximation. The left-side profiles were also shifted by 180°. For each muscle, the envelope was normalized to the median peak value calculated across cycles during the 30-RPM trial; this cadence was chosen as the reference since it was the easiest to maintain for patients.

The variance ratio (VR)^12^ was used to assess the intra-individual variability of the EMG envelopes for each muscle and subject:

1 $${\text{VR}} = \frac{{\mathop \sum \nolimits_{i = 1}^{K} \mathop \sum \nolimits_{j = 1}^{N} \frac{{x_{ij} - \overline{x}_{i} }}{{K \left( {N - 1} \right)}}}}{{\mathop \sum \nolimits_{i = 1}^{K} \mathop \sum \nolimits_{j = 1}^{N} \frac{{x_{ij} - \overline{x} }}{KN - 1}}}\quad {\text{with}}\quad \overline{x} = \frac{1}{K} \mathop \sum \limits_{i = 1}^{K} \overline{x}_{i}$$

where *K* = 360 is the number of samples, *N* = 30 is the number of cycles, *x* denotes the EMG envelope of a muscle, and $\overline{{x}}_{i}$ represents the mean value at the *i*-*th* sample over the *N* cycles. The VR indicates the overall variation of the data with respect to the mean profile: the higher is the value of VR, the higher is the intra-individual variability.

To address the first aim of the study (i.e., to investigate the modular muscle coordination during cycling) muscle synergies were extracted separately for each side and trial by applying nonnegative matrix factorization (NNMF)^20^,^29^ to the matrix *M*_(10,800×9)_ containing the EMG envelope of the 30 cadence-matched cycles for the 9 recorded muscles.^16^ NNMF decomposes *M* into the form ***M*** ≈ ***WH***, where *W*_9×S_ is the matrix of the synergy vectors containing the spatial information of muscle co-activation, *H*_S×10,800_ is the matrix of the synergy activation coefficients containing the temporal information of module recruitment, and *S* is the number of independent modules to be specified before NNMF application. One to nine synergies were extracted for each subject and trial, and *S* was chosen as the smallest number of synergies able to account for at least 90% of data variability evaluated in terms of variance accounted for (VAF):^4^,^16^

2 $${\text{VAF}} = 1 - \frac{{\mathop \sum \nolimits_{i = 1}^{9} \mathop \sum \nolimits_{j = 1}^{N} \left( {M_{ij} - R_{ij} } \right)^{2} }}{{\mathop \sum \nolimits_{i = 1}^{9} \mathop \sum \nolimits_{j = 1}^{N} \left( {M_{ij} } \right)^{2} }}$$

where *R* = *WH* is the matrix resulting from the reconstruction with the specified number of synergies and *N* = 10,800 is the number of samples.

#### Reconstruction of Muscle Activation Patterns with Healthy Modules

Regardless of the actual number of synergies required to account for our VAF criterion, in order to characterize the spatial module composition of the healthy group, the same most representative number of synergies was extracted for all healthy subjects and trials^11^ and the matrix *W*_HEALTHY_ was computed by averaging all of the obtained set of synergies.

To address the second aim of the study (i.e., to determine whether the spatial composition was preserved soon after stroke), the nonnegative reconstruction (NNR)^13^ algorithm was applied to the EMG envelopes of each trial by fixing the synergy vectors as *W*_HEALTHY_ and letting only the synergy activation coefficients *H* update at every algorithm iteration, according to the following multiplicative update rule:^20^

3 $$H_{\text{rc}} \leftarrow H_{\text{rc}} \frac{{\left( { W_{\text{HEALTHY}}^{T} M} \right)_{\text{rc}} }}{{\left( { W_{\text{HEALTHY}}^{T} W_{\text{HEALTHY}} H} \right)_{\text{rc}} }}$$

where r and c represent the rows and columns of the defined matrices, respectively, and the apex *T* denotes the transposed matrix. The VAF values of the reconstructed EMG were used to evaluate the success of this analysis.

Each vector of *W*_HEALTHY_ was normalized to unit norm before applying NNR, so that we could provide a rough estimate of the amount of activation of each module by comparing the *H* component.

#### Force Signal Analysis

The force signals were analyzed over the same 30 cadence-matched cycles used for the EMG analysis. Starting from the tangential ($F_{t }$) and the radial component ($F_{r }$), the total force was computed as:

4 $$F_{\text{total}} = \sqrt {F_{t}^{2} + F_{r}^{2} }$$

As for EMG envelopes, both the tangential and total force were expressed as function of the crank angle and re-sampled on a 360-point vector by means of a cubic spline approximation. Then, the left-side profiles were shifted by 180°.

The tangential force was analyzed also during the initial passive phase of each trial. Ten cycles during which the subjects were actually relaxed (assessed by visually inspecting the EMG signals) were extracted and expressed as function of the crank angle. An estimate of the active contribution provided by the patient alone, thus removing inertial and gravitational components, was computed as follows:

5 $$\Delta F_{t,j}(i) = \frac{1}{30} \mathop \sum \limits_{n = 1}^{30} \left. {F_{t, j}^{\text{active}}} \right.(i,n) - \frac{1}{10} \mathop \sum \limits_{n = 1}^{10} \left. {F_{t, j}^{\text{passive}}} \right.(i,n) \quad {\text{with}}\quad j \in \left\{ {{\text{right}};{\text{left}}} \right\}$$

where $F_{t, j}^{\text{active}}$ and $F_{t, j}^{\text{passive}}$ represent the tangential force profiles computed during the active and the passive phase, respectively. The indexes *i* and *n* indicate the number of sample and cycle, respectively. This estimate was calculated both to remove the assistance provided by the motor and to compensate for potential differences of the passive components due to the different weight of the two legs after stroke.^2^

#### Cycling-Based Metrics

A set of metrics were extracted from the force profiles and the reconstructed synergy activation coefficients calculated for the 30-RPM trial, being this cadence the easiest to maintain for all patients.The work produced by each leg was computed as follows:^2^

6 $${\text{Work}}_{j} = \frac{1}{360} \mathop \sum \limits_{i = 1}^{360} \Delta F_{t,j} \left( i \right)\quad {\text{with}}\quad j \in \left\{ {{\text{right}};{\text{lef}}t} \right\}$$

To compare patients and healthy subjects in terms of tangential force profile, the area symmetry index (ASI) was defined as:^22^

7 $${\text{ASI}} = 1 - \frac{{\mathop \sum \nolimits_{i = 1}^{360} \left| {\Delta F_{{t,{\text{aff}}}} \left( i \right) - \Delta F_{{t,{\text{healthy}}}} \left( i \right)} \right|}}{{\mathop \sum \nolimits_{i = 1}^{360} \Delta F_{{t,{\text{aff}}}} \left( i \right) + \mathop \sum \nolimits_{i = 1}^{360} \Delta F_{{t,{\text{healthy}}}} \left( i \right)}}$$

where $\Delta F_{{t,{\text{aff}}}}$ represents the active contribution of the affected leg and $\Delta F_{{t,{\text{healthy}}}}$ is computed by averaging both sides of all healthy subjects. This index ranges from 0 to 1 (i.e., complete superposition of the two profiles).

The index of effectiveness (IE)^13^ was also computed for each side (*j*) of all subjects as:

8 $${\text{IE}}_{j} = \frac{{\mathop \sum \nolimits_{i = 1}^{360} F_{t,j} \left( i \right)}}{{\mathop \sum \nolimits_{i = 1}^{360} F_{{{\text{total}},j}} \left( i \right)}} \quad {\text{with}}\quad j \in \left\{ {{\text{right}};{\text{lef}}t} \right\}$$

IE ranges from 0 to 1 (i.e., the whole applied force is used to propel the crank, with no dissipation towards the radial direction).

Finally, the shape symmetry index (SSI)^22^ was computed to compare the two groups of subjects in terms of synergy activation coefficients:

9 $${\text{SSI}}_{j} = \frac{{C_{{h_{{{\text{aff}},j}} h_{{{\text{healthy}},j}} }} }}{{\sqrt {\mathop \sum \nolimits_{i = 1}^{360} h_{{{\text{aff}},j}}^{2} \left( i \right)\mathop \sum \nolimits_{i = 1}^{360} h_{{{\text{healthy}},j}}^{2} \left( i \right)} }}$$

where $h_{aff,j}$ represents the reconstructed activation coefficient of the affected leg of each patient for the *j*-*th* synergy, $h_{{{\text{healthy}},j}}$ is the reconstructed activation coefficient for the corresponding synergy achieved by averaging all healthy subjects (both sides), and $C_{{h_{aff,j} h_{{{\text{healthy,}}j}} }}$ is the circular cross-correlation function at lag 0. SSI ranges from −1 to 1 (i.e., identical profiles shape regardless differences in amplitude).

### Statistical Analysis

In order to justify the existence of the matrix *W*_HEALTHY_, a repeated-measures two-factor ANOVA was applied to the all the synergy vectors (i.e., each element of the matrix *W*_*mxs*_, where *m* indicates the muscle and *s* the synergy vector) extracted from the healthy subjects among different cadences and sides (dominant/non-dominant).

The Variance Ratio of each muscle, the angular peak positions of the reconstructed synergy activation coefficients and the angular peak position of the tangential force profiles underwent a mixed two-factor ANOVA to compare different cadences (20–50 RPM; within-subject factor) and legs (dominant/non-dominant for healthy and affected/unaffected for patients; between-subject factor).

The Spearman’s correlation coefficient between the number of modules extracted from the patients’ affected leg and the MI score was computed.

Finally, the correlations between the cycling-based metrics and the gait parameters were evaluated by computing the Pearson’s correlation coefficient.

## Results

Twelve healthy subjects (8 males, mean age of 68 ± 5 years) and 16 stroke patients (11 males, mean age of 70 ± 10 years) completed the experimental session. Patients were characterized by a wide range of motor impairment, with the MI score and the gait speed ranging from 52 to 91 and from 0.37 to 1.18 m/s, respectively (Table 1).

**Table 1:** Clinical and demographic details of the post-stroke patients.

ID	Sex	Age (years)	Days post-stroke	Type of stroke	Paretic side	Motricity index, leg [0–100]	Gait speed (m/s)	Gait assistance
P1	M	75	90	Ischemic	Right	52	0.37	Cane
P2	M	81	120	Ischemic	Left	83	0.58	None
P3	M	70	100	Ischemic	Left	83	0.69	None
P4	M	79	60	Ischemic	Left	75	0.61	Therapist
P5	M	57	16	Ischemic	Right	83	0.96	None
P6	M	66	110	Ischemic	Left	91	0.95	None
P7	M	68	106	Hemorrhagic	Right	58	1.01	None
P8	M	72	19	Ischemic	Left	75	0.39	Therapist
P9	M	74	17	Ischemic	Left	75	1.18	None
P10	F	47	9	Ischemic	Left	52	0.44	None
P11	M	73	18	Ischemic	Right	75	0.79	None
P12	M	82	10	Ischemic	Right	75	0.48	Therapist
P13	F	76	12	Hemorrhagic	Left	75	0.77	Therapist
P14	F	58	18	Hemorrhagic	Left	63	0.82	None
P15	F	84	15	Ischemic	Left	63	0.63	Supervision
P16	F	65	78	Ischemic	Right	69	0.52	Supervision

### Muscle Synergies

The EMG intra-individual variability, averaged across subjects and cadences, was always lower than 0.37, with RF and TA showing the lowest and highest VR values, respectively. A significant effect of cadence was found only for Gmax, while a significant effect of leg was revealed for BFlh and BFsh (Table S1).

**Figure 2:**
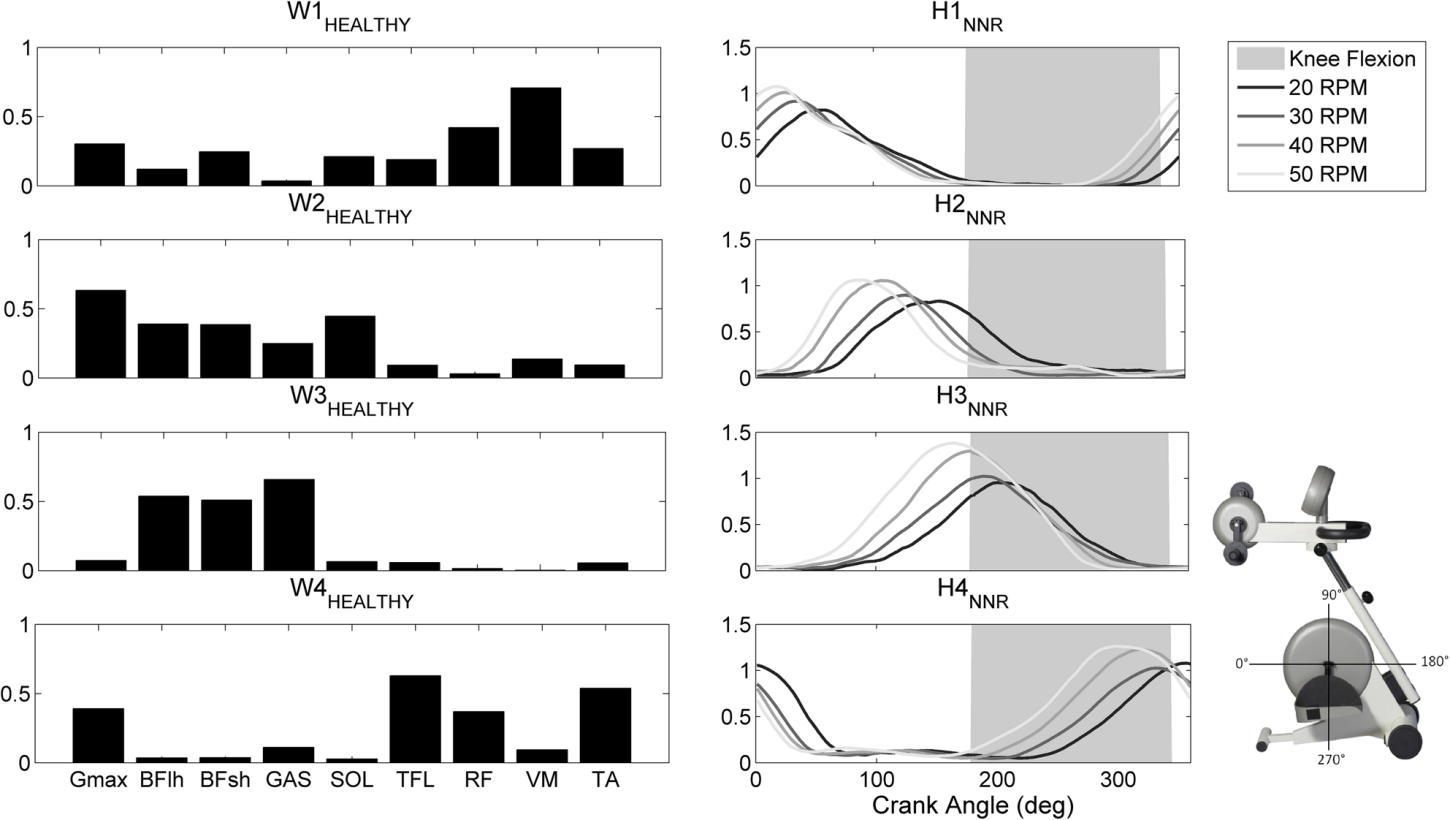
Average set of synergies extracted from the healthy subjects (*W*_HEALTHY_, left column) and reconstructed synergy activation coefficients at different cadences, obtained from the application of the Nonnegative Reconstruction by fixing the matrix *W*_HEALTHY_ (right column, each line is obtained by averaging both sides of all subjects).

The number of synergies extracted from the healthy participants varied between 2 and 5: among 96 total extractions (12 subjects, 2 legs, 4 cadences), 2% required two modules, 25% three modules, 60% four modules, and 13% five modules. Thus, to characterize the module composition of the healthy subjects, four synergies were extracted from all trials, and the obtained synergy vectors were compared. Since no major significant differences were highlighted in the spatial composition of the healthy subjects (Table S2), the matrix *W*_HEALTHY_ was used to reconstruct the activation timing of the healthy population (Fig. 2). The synergy S_1_ co-activates the knee extensors (VM and RF) and is mainly responsible for power production; the synergy S_2_ is mainly composed by the activation of Gmax and SOL, with some contribution of the hamstring muscles, and assists the transition between knee extension and flexion; the synergy S_3_ is composed by the activation of mono-articular (BFsh) and bi-articular (BFlh and GAS) knee flexors, and aids limb recovery during flexion; finally, the synergy S_4_ works in the last part of the pedaling cycle and co-activates ankle dorsal-flexors (TA) and hip flexors (TFL and RF). The application of the NNR led to high VAF values (VAF_20RPM_ = 84 ± 6%, VAF_30RPM_ = 87 ± 4%, VAF_40RPM_ = 85 ± 5%, VAF_50RPM_ = 81 ± 10%).

**Figure 3:**
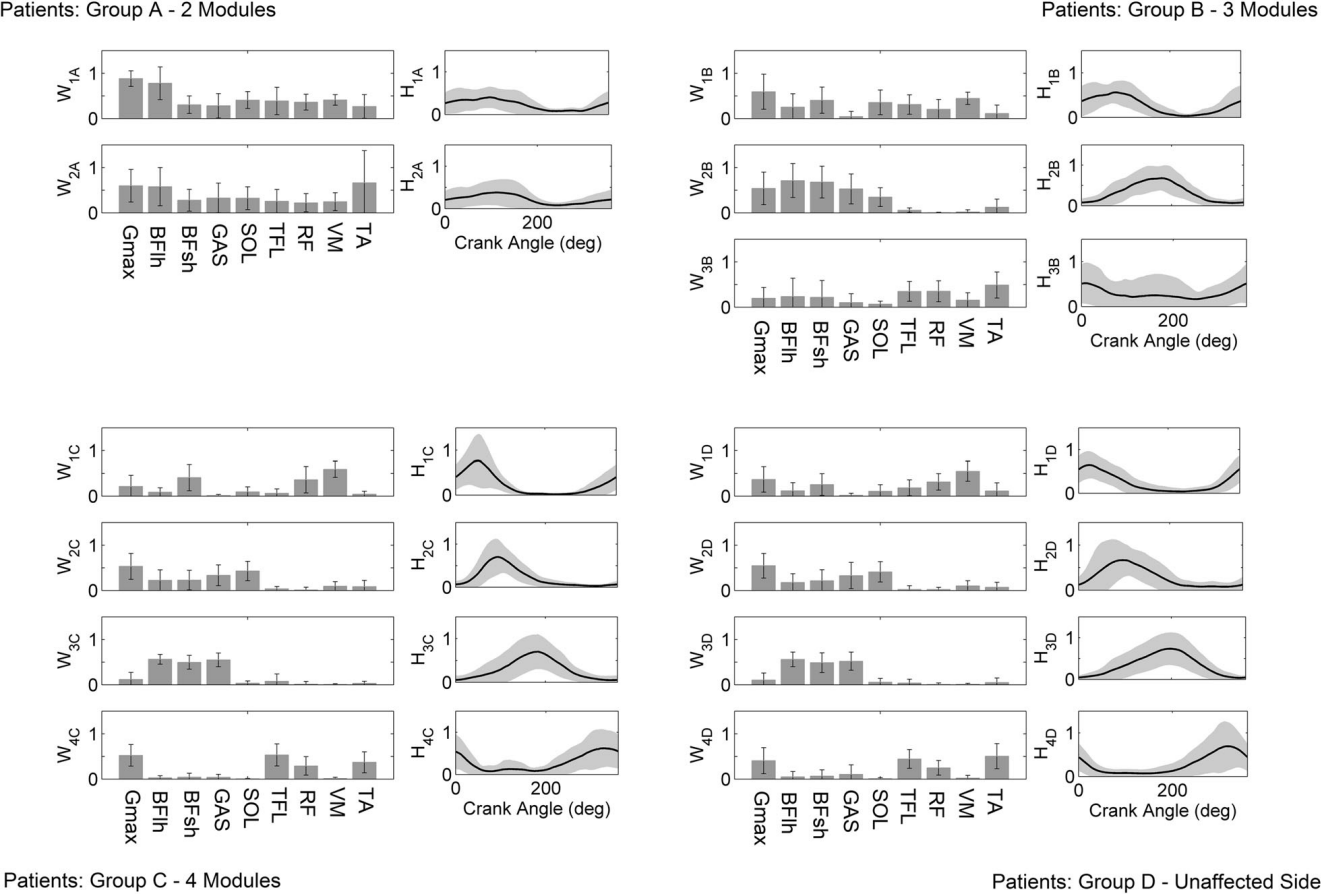
Spatio-temporal structure of the modules extracted from the patients. Group A, B, and C represent the average among subjects with two (*n* = 3), three (*n* = 6), and four (*n* = 7) modules in the affected side; Group D shows the average among all subjects with 4 modules (*n* = 16) in the unaffected side. Synergy vectors *W* and activation coefficients *H* obtained at different cadences are also averaged.

When NNMF was applied on patients’ data, four synergies were needed to account for cycling muscle activity in the unaffected leg in 76% of trials; thus, as for healthy, 4 synergies were extracted from all trials and subjects. When taking into account the affected legs, the majority of patients (*n* = 7) required four modules, six of them required three modules, and three of them just two modules. When a different number of synergies was needed to explain data variability at different cadences, the most representative number was extracted for all trials of that patient. Figure 3 shows the module composition and the activation timing of each module averaged for each sub-group of patients. The lack of timing independence in the two-module sub-group resulted from averaging few data (*n* = 3) with a high inter-subject variability (see Supplementary File S1 for subject-specific module composition).

The correlation between the number of modules extracted from the affected leg and the MI score was significant (*p* = 0.039), with a Spearman’s correlation coefficient of 0.521.

**Figure 4:**
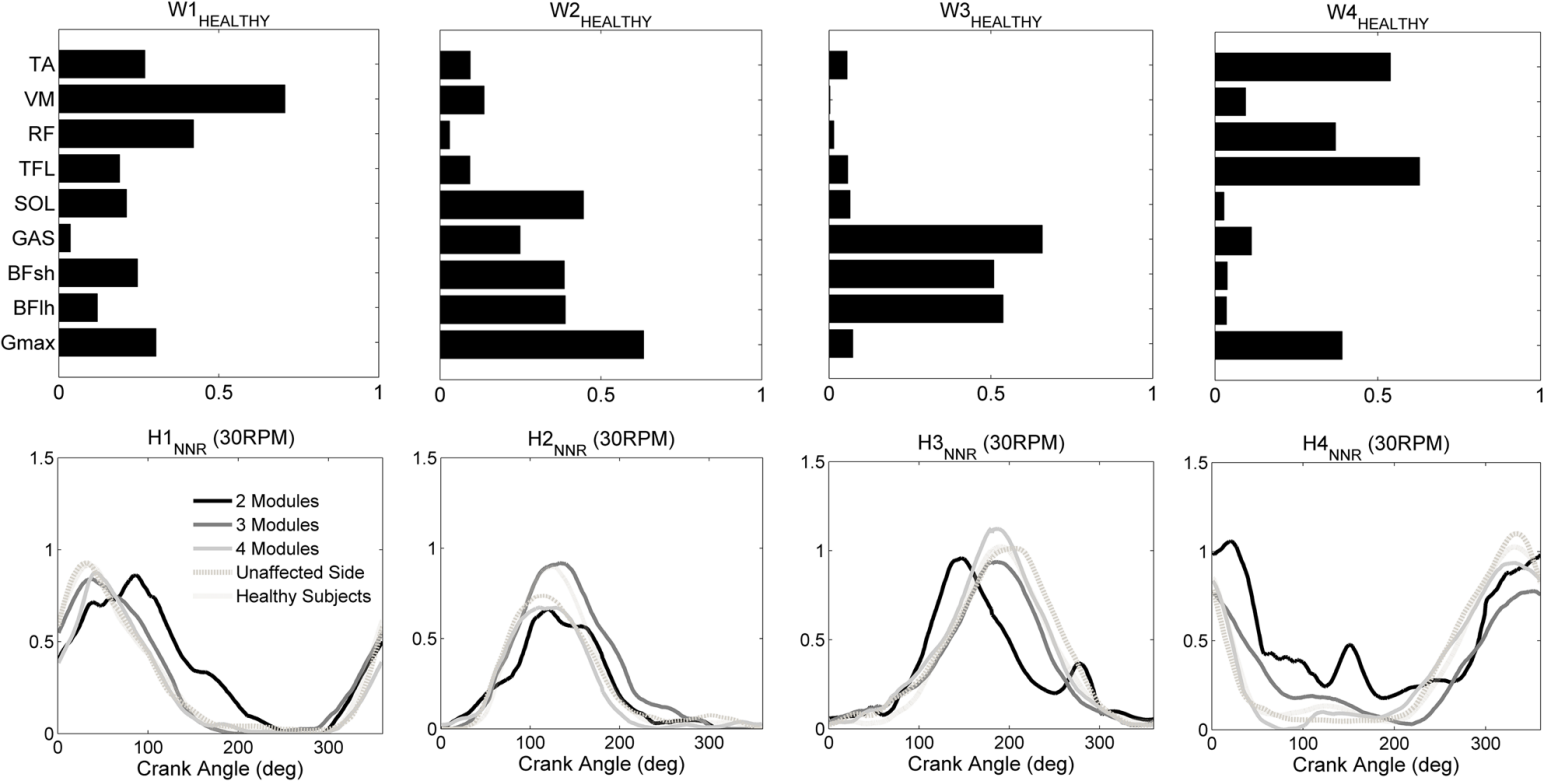
Reconstructed module recruitment (synergy activation coefficients *H*, lower panels) by applying NNR with fixed *W*_HEALTHY_ (upper panels) for the two-, three-, and four-module affected sub-groups, for the unaffaced sub-group, and for the healthy subjects group at 30 RPM.

Regardless the number of extracted modules, the matrix *W*_HEALTHY_ was used to reconstruct the muscle coordination of both sides of all patients. High VAF values were obtained from the reconstruction of both the unaffected (VAF_20RPM_ = 86 ± 5%, VAF_30RPM_ = 87 ± 6%, VAF_40RPM_ = 84 ± 8%, VAF_50RPM_ = 87 ± 7%) and affected side (VAF_20RPM_ = 82 ± 14%, VAF_30RPM_ = 86 ± 7%, VAF_40RPM_ = 86 ± 6%, VAF_50RPM_ = 85 ± 7%). Figure 4 shows the reconstructed synergy activation coefficients averaged over the affected side of patients with two, three, and four modules, the unaffected side of all patients, and both sides of healthy subjects, for the 30-RPM trials.

Table 2 reports the angular peak positions of the reconstructed synergy activation coefficients averaged over the healthy dominant/non-dominant leg and the patients affected/unaffected side. For all activation coefficients the peak was generated significantly earlier at higher speed.

**Table 2:** Mean values (standard deviation) of the angular peak positions of the reconstructed synergy activation coefficients (H1_NNR_–H4_NNR_) and of the tangential force profile ($F_{t}$).

	20 RPM [deg]	30 RPM [deg]	40 RPM [deg]	50 RPM [deg]	Cadence effect	Leg effect
H1_NNR_						
Dominant leg	44 (20)	26 (18)	11 (18)	5 (19)	<0.001*	0.033**
Non-dominant leg	53 (19)	41 (12)	32 (15)	22 (11)		
Unaffected leg	52 (17)	35 (17)	25 (14)	12 (20)		
Affected leg	63 (24)	41 (27)	30 (20)	17 (21)		
H2_NNR_						
Dominant leg	156 (40)	123 (17)	119 (43)	105 (41)	<0.001*	0.510
Non-dominant leg	131 (23)	125 (20)	106 (22)	84 (17)		
Unaffected leg	137 (34)	122 (23)	98 (25)	87 (34)		
Affected leg	145 (31)	125 (23)	108 (31)	91 (21)		
H3_NNR_						
Dominant leg	219 (21)	194 (25)	189 (31)	180 (33)	<0.001†	0.001‡
Non-dominant leg	198 (16)	181 (15)	168 (16)	155 (21)		
Unaffected leg	202 (44)	188 (44)	189 (19)	176 (28)		
Affected leg	186 (30)	174 (29)	166 (25)	152 (21)		
H4_NNR_						
Dominant leg	349 (7)	309 (63)	315 (17)	306 (20)	<0.001†	0.117
Non-dominant leg	2 (20)	335 (25)	328 (25)	307 (27)		
Unaffected leg	348 (26)	328 (23)	315 (25)	296 (21)		
Affected leg	20 (63)	344 (40)	341 (55)	310 (38)		
$$\varvec{F}_{\varvec{t}}$$						
Dominant leg	154 (7)	159 (5)	163 (8)	167 (9)	<0.001^||^	0.001¥
Non-dominant leg	139 (9)	140 (7)	142 (7)	146 (7)		
Unaffected leg	147 (13)	152 (13)	152 (13)	156 (12)		
Affected leg	146 (14)	150 (13)	151 (14)	151 (15)		

Results of the mixed two-factor ANOVA are also reported

*The *post hoc* analysis revealed that all cadences were significantly different from the others

^†^The *post hoc* analysis revealed that 20 RPM was significantly different from all the other cadences; 30 and 40 RPM were significantly different from 50 RPM

^||^The *post hoc* analysis revealed that all cadences were significantly different from the others, but 30 from 40 RPM

**The *post hoc* analysis revealed that the healthy dominant leg was significantly different from the patients affected leg

^‡^The *post hoc* analysis revealed that the healthy dominant and the patients unaffected leg were significantly different from the patients affected leg

^¥^The *post hoc* analysis revealed that the healthy dominant leg was significantly different from the healthy non-dominant leg

### Pedal Forces

**Figure 5:**
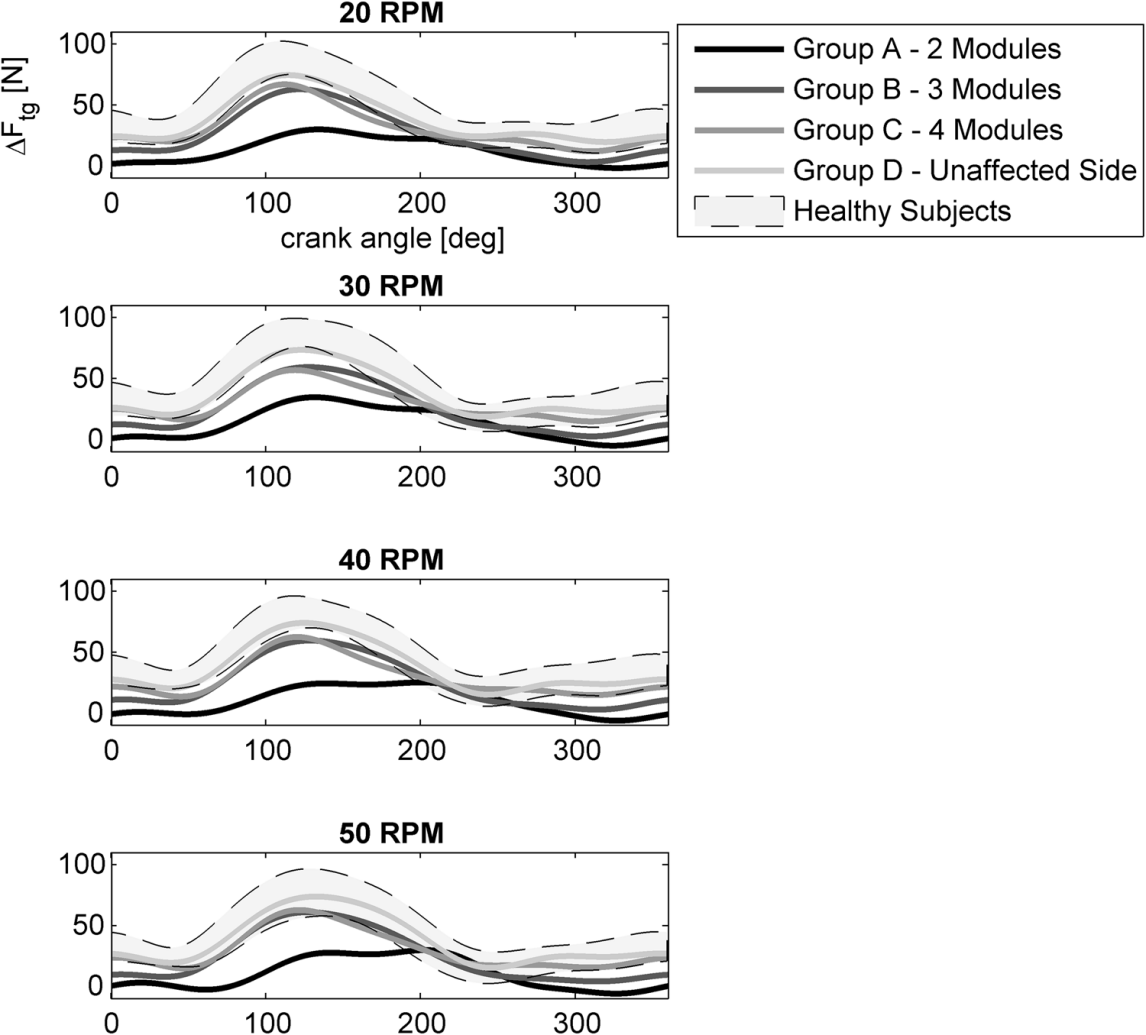
Tangential force profiles without passive contributions for the different sub-groups of patients (the solid lines represent the mean value of the corresponding sub-group) and for the healthy subjects group (the area indicate the mean value ± the standard deviation).

Figure 5 shows the mean active tangential force profiles of the affected side for the two-, three-, and four-module sub-group, the profile of the unaffected leg averaged over all patients, and the normality range (mean ± standard deviation of healthy). In all profiles, the force peak well corresponded to the activity of the knee-extensor muscles during the down-stroke phase of the pedaling cycle. A smaller and delayed force output was observed for the affected leg of the two-module sub-group at all speeds. The affected leg of the patients with three and four modules produced a propulsive force just below the normality range, while the force of the unaffected leg was similar to the healthy subjects one.

Despite the phase-advance of the EMG activation timing, the peak pedaling force was generated significantly later at higher speed (Table 2).

### Cycling-Based Metrics and Gait Parameters

**Figure 6:**
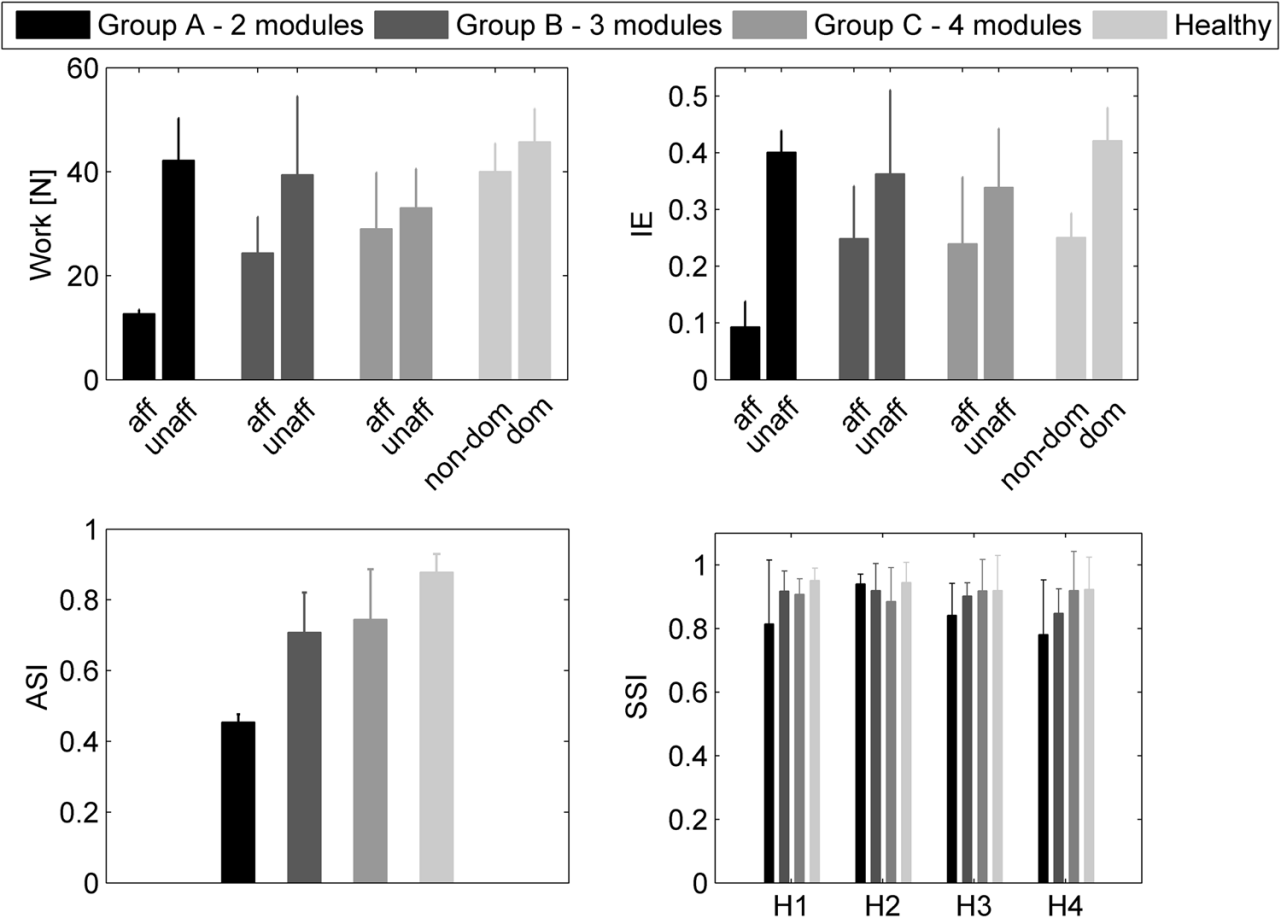
Cycling-based metrics computed for the trial at 30 RPM. Mean values and standard deviation are reported for the two-, three-, and four-module sub-group of patients and for the healthy subjects group.

Figure 6 reports the cycling-based metrics for the three sub-groups of patients and for the healthy subjects calculated for the 30-RPM trials. The work produced by the affected side increased with the number of modules, but remained always lower than the one of the unaffected side and of the healthy subjects. The index of effectiveness was unbalanced between the two sides for all subjects, with the two-module sub-group showing the highest unbalance, due to a very low effectiveness of the affected side. A low superposition between the affected leg and the healthy force profiles was observed for the low-complexity patients and the ASI increased with the number of modules. Analogously, the similarity in shape between synergy activation coefficients of patients and healthy subjects (SSI) was lower for the two-module sub-group, except for the second synergy, which did not show a clear trend.

**Figure 7:**
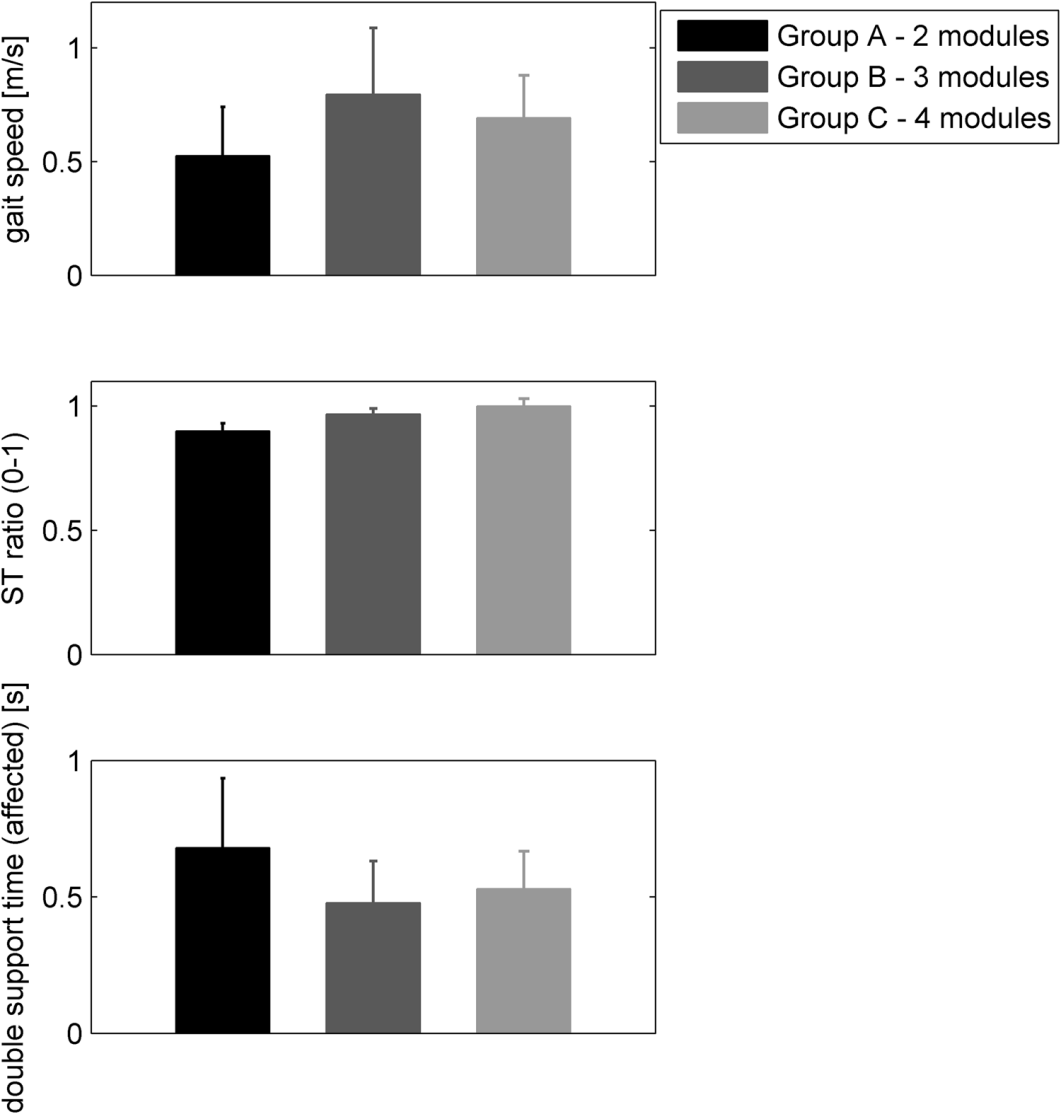
Gait parameters computed for the two-, three-, and four-module sub-group of patients. Mean values and standard deviation are reported.

Figure 7 reports the gait parameters for the three sub-groups of patients. A clear trend towards higher scores for patients with an increasing number of modules was observed for the ST ratio. As for the gait speed and the double support time, the two-module sub-group showed the lowest and the highest values, respectively.

A moderate correlation was found between some of the cycling-based metrics and some gait parameters, as reported in Table 3.

**Table 3:** Correlations between cycling-based metrics (at 30 RPM) and gait parameters

	Gait speed	ST ratio	Double support time (affected)
Work_affected_	0.513	0.624	–
IE_affected_	–	–	–
ASI	0.555	0.653	−0.511
SSI_H1_	–	–	–
SSI_H2_	–	–	–
SSI_H3_	–	0.546	−0.589
SSI_H4_	–	0.716	–

Only significant Pearson’s correlation coefficient (*p* < 0.05) are reported

## Discussion

Our findings demonstrated that the CNS relies on a low–dimensional modular organization to control recumbent pedaling both in healthy elderly subjects and post-acute stroke patients. Healthy elderly subjects share a common motor control strategy based on four muscles synergies, whose spatial composition was consistent between legs and at different cadences. The identified muscle synergies well correspond to the four biomechanical functions previously proposed.^28^ The four synergies can be arranged into two pairs: one pair consists of the first (knee extensors) and third synergy (knee flexors) and produces the energy needed to propel the crank during limb extension and flexion, respectively; the other pair involves the second (hamstrings, ankle plantarflexors, and gluteus) and fourth synergy (RF, TFL and ankle dorsiflexors) and facilitates the energy transfer to the crank near the end of extension and flexion, respectively, as well as assists limb transition from extension to flexion and* vice versa*.

The structure of the identified modules is highly similar to that identified during upright pedaling in young healthy subjects,^12^ while only three muscle synergies were extracted in other two studies, which investigated muscle coordination underlying recumbent pedaling in young healthy subjects^4^ and upright pedaling in professional cyclists.^16^ Two of these synergies well correspond to S_1_ and S_4_ identified in our study, while the remaining one can be interpreted as a merging of S_2_ and S_3_. The smaller number of synergies can be explained by the higher power outputs produced by younger and more trained subjects, which may result in a higher signal-to-noise ratio, and therefore in a higher VAF explained by fewer synergies.^34^ Another possible explanation is the lower pedaling rate of our study. Indeed, leg muscles behave differently at different cadences, with thigh muscles exhibiting an earlier activation as pedaling rate increases, and the soleus muscle showing a small trend towards a later shift.^26^ This behavior might result in the merging of our second and third synergies. Despite the differences in dimensionality, our results confirm that the cycling modular control of healthy individuals is not influenced by pedaling position^16^ and aging,^25^ being the biomechanical constraints similar.^35^

We found a moderate correlation between the MI score and the number of modules extracted from the patients’ affected side. This result confirms that, as previously found for walking,^8^,^11^ also for cycling the higher is the level of impairment the lower is the motor coordination complexity. Patients who exhibited less than four modules did not share a common modular organization (see supplementary file S1). When four modules were extracted also for the affected side, a modular organization similar to the age-matched controls was observed. Finally, as expected, the unaffected side shared a common modular organization with controls, suggesting the absence of compensatory strategies in the early phase after stroke.

Despite the number of extracted modules, the healthy modules were able to reconstruct the muscle coordination of both sides of the patients group (mean VAF values >82%), suggesting that the spatial composition was preserved during pedaling soon after stroke. Thus, since a reduced number of modules was extracted for the affected side of the most impaired patients but the spatial composition was anyhow preserved, we concluded that deficits in motor control could be ascribed to alterations in the temporal recruitment of the healthy modules, probably due to a lack of independence in the corticospinal drive.^35^ This result was similar to what observed during walking for chronic stroke patients.^11^ Conversely, when muscle coordination was investigated during walking in subacute stroke patients,^15^ activation signals but not motor modules were preserved after stroke, and the Authors suggested that the changes in modularity were due to compensatory strategies aimed at balancing strength loss in the paretic side. Since during cycling postural control is not required, the need for compensatory strategy is reduced, thus providing a possible explanation for our different result.

Comparing the reconstructed synergy activation coefficients between healthy subjects and stroke patients (Fig. 4), we observed a prolonged excitation of the knee extensors (H1) and a phase-advanced excitation of the hamstrings muscles (H3) in the low-complexity sub-group, in line with the types of abnormalities previously found from single-muscle EMG analysis.^18^

Consistent with the literature,^16^,^26^ an earlier activation of motor modules was found as the pedaling rate increased (Table 2). This mechanism, known as activation dynamics, is a compensatory strategy aimed at maintaining the same force profile with respect to the pedaling cycle despite the constant electromechanical delay.^26^ However, as already observed,^16^ this compensatory strategy was not sufficient to fully prevent a small forward shift at high pedaling rates.

Similar to what already observed for walking,^11^ the reduced coordination complexity after stroke worsens the biomechanical performance of pedaling, as highlighted by the lower work production and mechanical effectiveness of the affected side in the two-module sub-group of patients (Fig. 6). This suggests that a reduced number of extracted modules may excessively constrain the motor output and alter the execution of the primary biomechanical functions.

We also observed that patients who exhibited a low-complexity motor coordination during pedaling, walked slower, with a more asymmetrical stance time, and a prolonged double support time in the affected leg (Fig. 7). This result supports the hypothesis of a shared modular control between cycling and pedaling.

We found significant correlations between cycling-based metrics and gait parameters (Table 3), suggesting that neurophysiological and biomechanical quantities of cycling performance can inform on walking dysfunctions. Specifically, the work produced by the paretic leg was positively correlated with the gait speed and the ST ratio, suggesting that the higher is the strength in the paretic side, the more efficient, secure and symmetrical is the walking pattern. Moreover, the alterations of the affected side in the recruitment of the two synergies characterizing the up-stroke phase of the pedaling cycle (S_3_ and S_4_) were positively correlated with the ST ratio. These two cycling synergies share a similar muscle composition with the walking synergies responsible for leg deceleration during late swing (S_3_) and ground clearance of the foot during early swing (S_4_).^27^ Thus, alterations of these cycling synergies may correspond to an impaired muscle coordination during the swing phase of gait, resulting in a prolonged swing time and a corresponding reduced stance time (low ST ratio) of the paretic leg.

The study might have some methodological limitations. EMG data of different subjects may be affected by different levels of signal-to-noise ratio, which can alter the number of extracted modules. However, we found that the intra-individual variability was overall similar among patients and controls (Table S1), suggesting a similar quality of the EMG signals. As done previously,^16^ the technique used to normalize the EMG envelopes did not allow to take into account the amplitude of the muscle activity for the comparison of muscle synergies. However, a standardized normalization technique able to correctly quantify the power output contribution from each muscle synergy does not exist yet.^16^ Another limitation is the absence of any dynamic measures able to assess the contribution of each leg to propulsion during walking that could be very important to strengthen the correlation analysis between cycling and walking.^7^

## Conclusions

A description of the modular control of cycling soon after stroke is provided: as expected, a common modular organization was shared between the unaffected side of the patients and the healthy volunteers, while a reduced complexity was observed for the affected side of the most impaired patients. Our findings provide supportive evidences to the hypothesis of a shared modular control between cycling and walking,^4^ further promoting the use of pedaling as a rehabilitation method as well as an assessment tool,^3^ mainly in the early phase after stroke when patients can be still unable to perform a safe and active gait training. In this framework, synergy analysis can be used both to quantify motor recovery during the rehabilitation process, and to define novel interventions, e.g., based on functional electrical stimulation, aimed at driving an altered modular control back to the healthy coordination. Furthermore, the proposed cycling-based indicators can help clinicians in providing a deep quantitative analysis of lower limb motor impairment after stroke.

## Electronic supplementary material

Below is the link to the electronic supplementary material.

Supplementary material 1 (PDF 3248 kb)
