# Supplementary material for: Neuro-Mechanics of Recumbent Leg Cycling in Post-Acute Stroke Patients
Source: Ann Biomed Eng. 2016 Jun 1;44(11):3238–51. doi: 10.1007/s10439-016-1660-0 (PMC5093201; doi:10.1007/s10439-016-1660-0)
Supplement: Supplementary file 1 — Supplementary material 1 (PDF 3248 kb) [file 10439_2016_1660_MOESM1_ESM.pdf]

Supplementary Material S1: Subject-specific extracted muscle synergies at different pedaling cadences (20,30,40 and 50 RPM). Each page contains the spatial composition of the extracted modules (W) and the temporal information regarding module recruitment (H) for a different patient. Patients' detailed information, including clinical scales and biomechanical measurements, are also reported.

PATIENT P1

Group: A – 2 modules

Age (years): 75

Days post Stroke: 90

Type of Stroke: ischemic

Motricity Index (leg subscale): 52

Gait Speed: 0.37 m/s

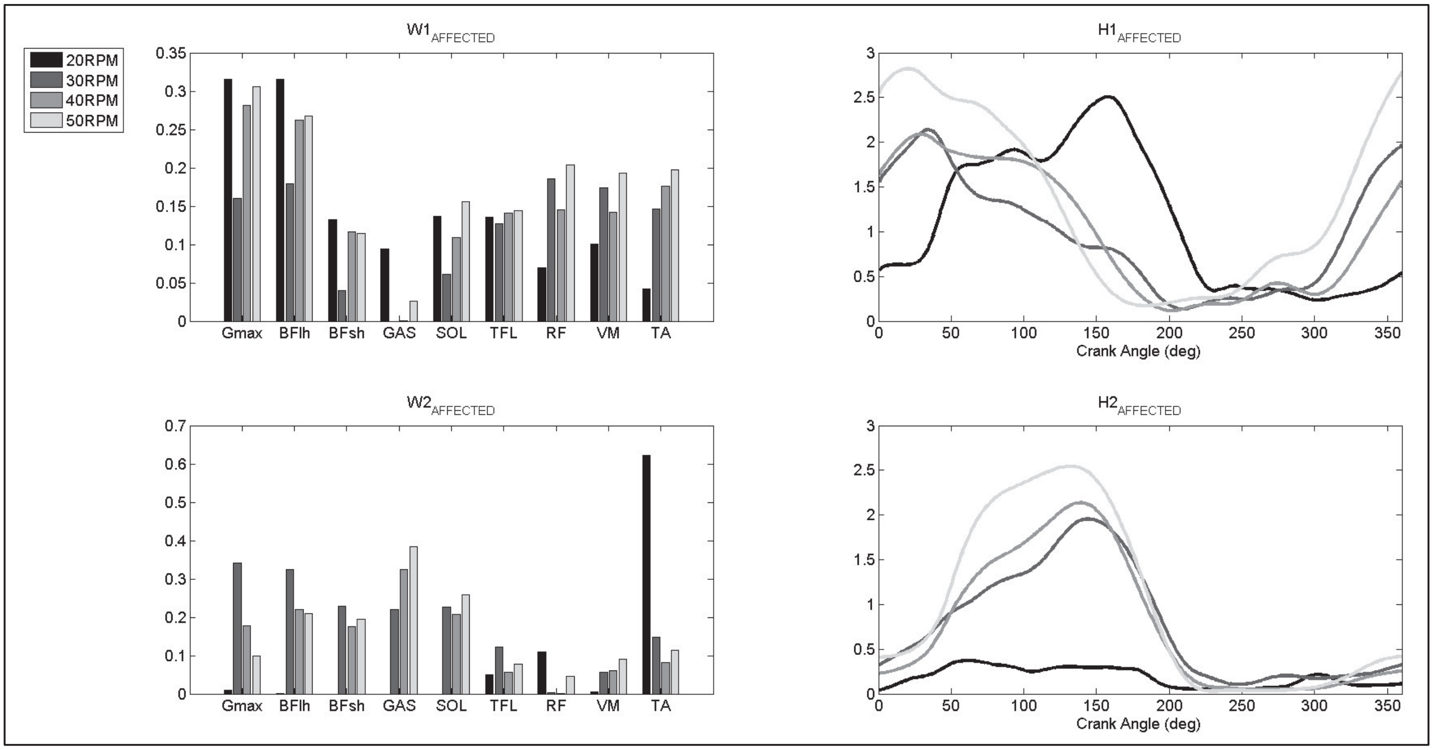

PATIENT P2

Group: C– 4 modules

Age (years): 81

Days post Stroke: 120

Type of Stroke: ischemic

Motricity Index (leg subscale): 83

Gait Speed: 0.58 m/s

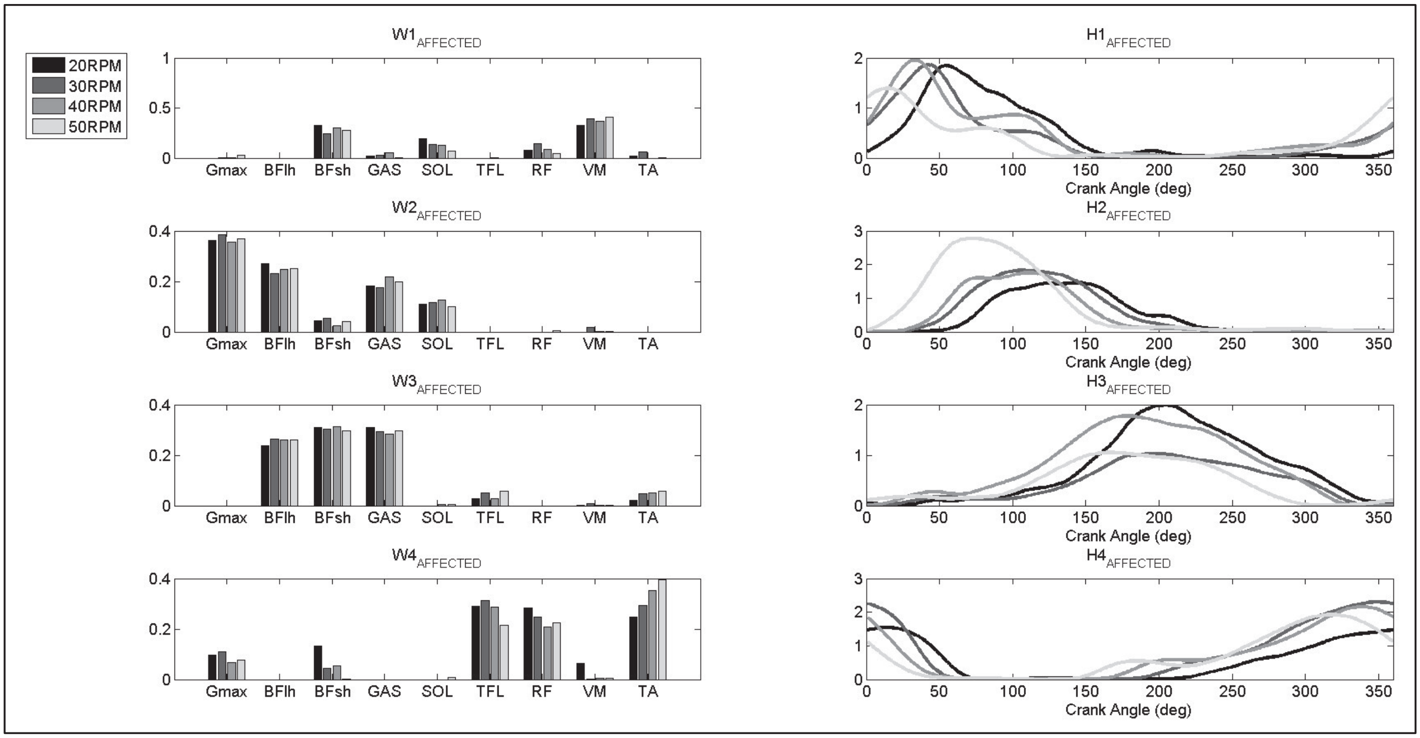

PATIENT P3

Group: C –4 modules

Age (years): 70

Days post Stroke: 100

Type of Stroke: ischemic

Motricity Index (leg subscale): 83

Gait Speed: 0.69 m/s

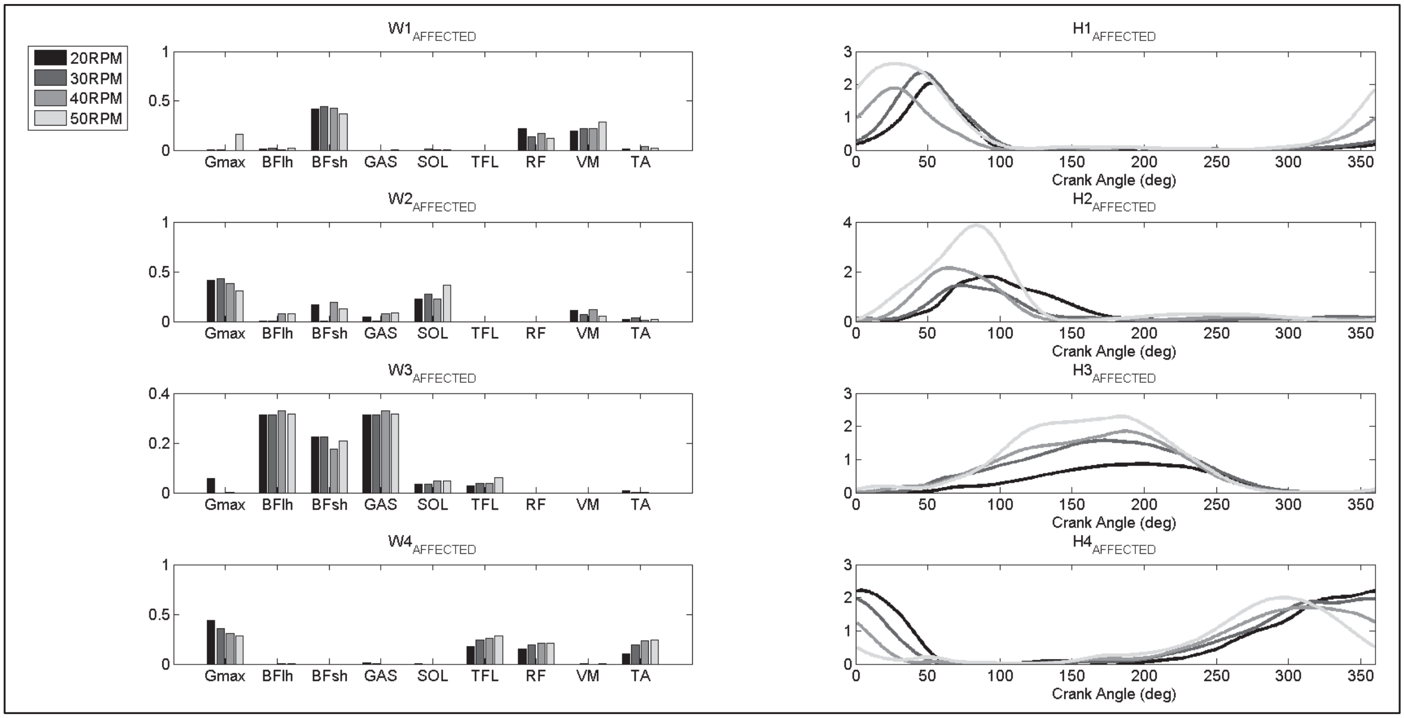

PATIENT P4

Group: C – 4 modules

Age (years): 79

Days post Stroke: 60

Type of Stroke: ischemic

Motricity Index (leg subscale): 75

Gait Speed: 0.61 m/s

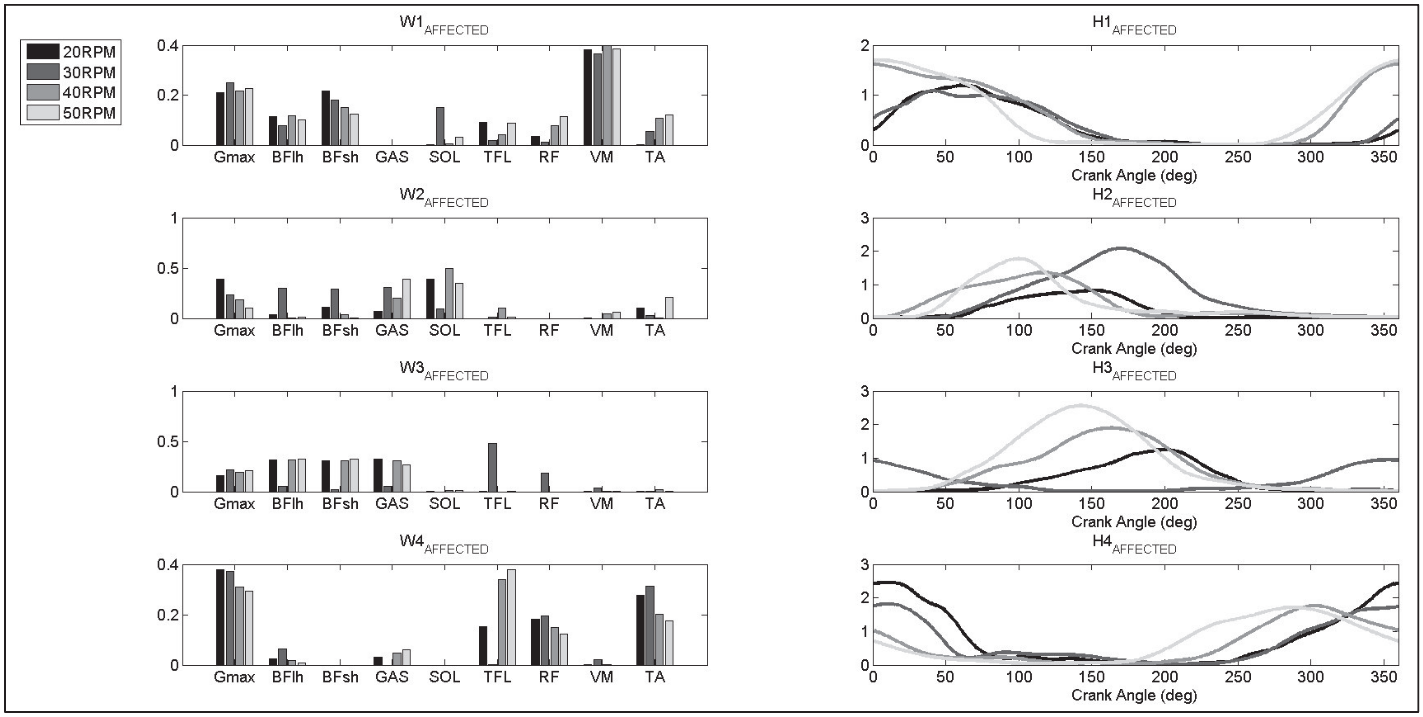

PATIENT P5

Group: C – 4 modules

Age (years): 57

Days post Stroke: 16

Type of Stroke: ischemic

Motricity Index (leg subscale): 83

Gait Speed: 0.96 m/s

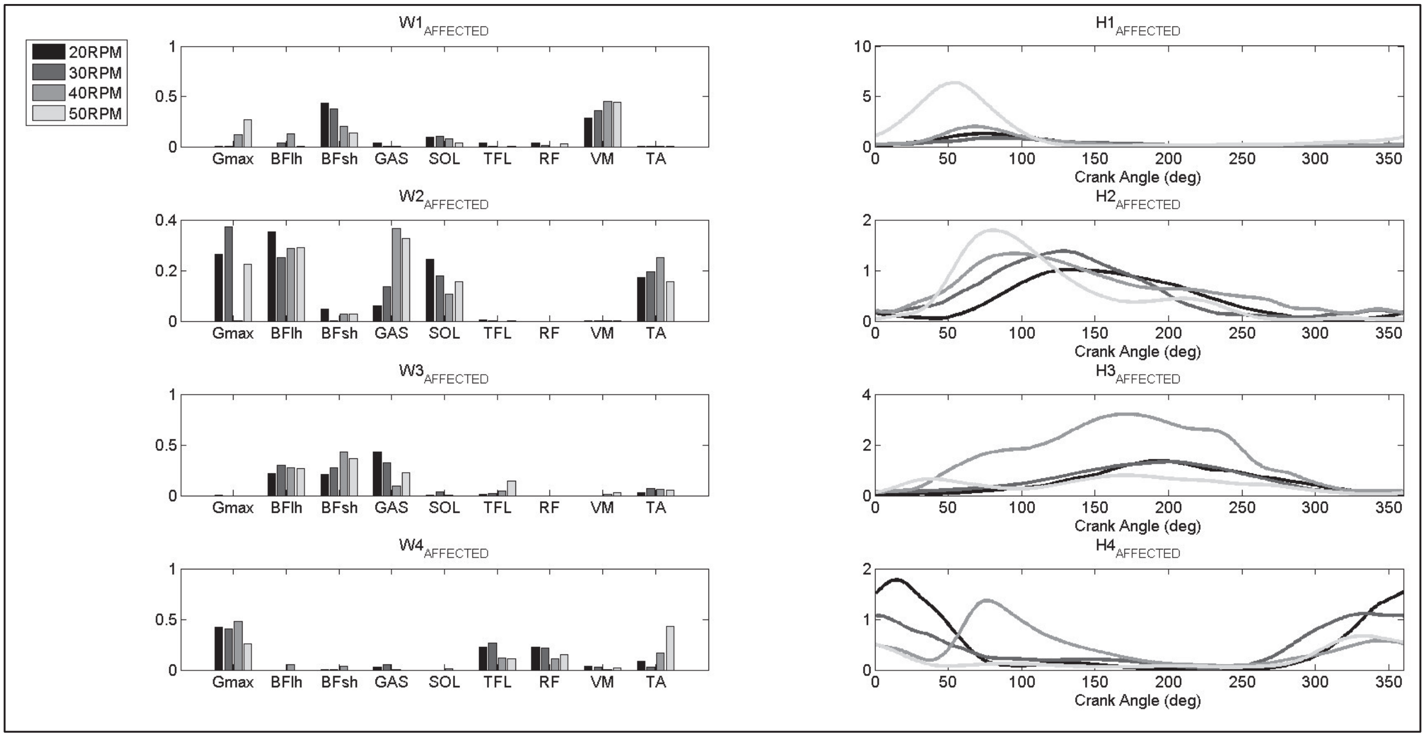

PATIENT P6

Group: B – 3 modules

Age (years): 66

Days post Stroke: 110

Type of Stroke: ischemic

Motricity Index (leg subscale): 91

Gait Speed: 0.95 m/s

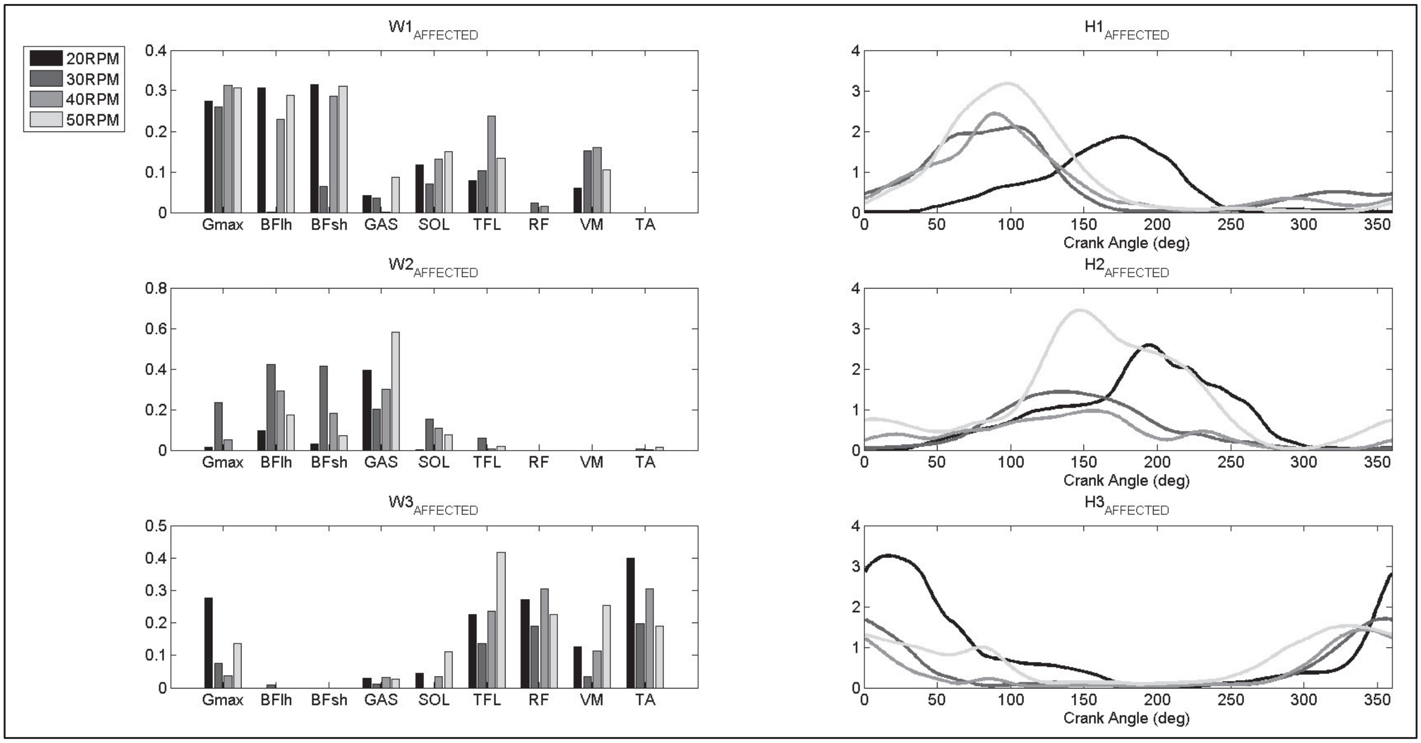

PATIENT P7

Group: B – 3 modules

Age (years): 68

Days post Stroke: 106

Type of Stroke: hemorragic

Motricity Index (leg subscale): 58

Gait Speed: 1.01 m/s

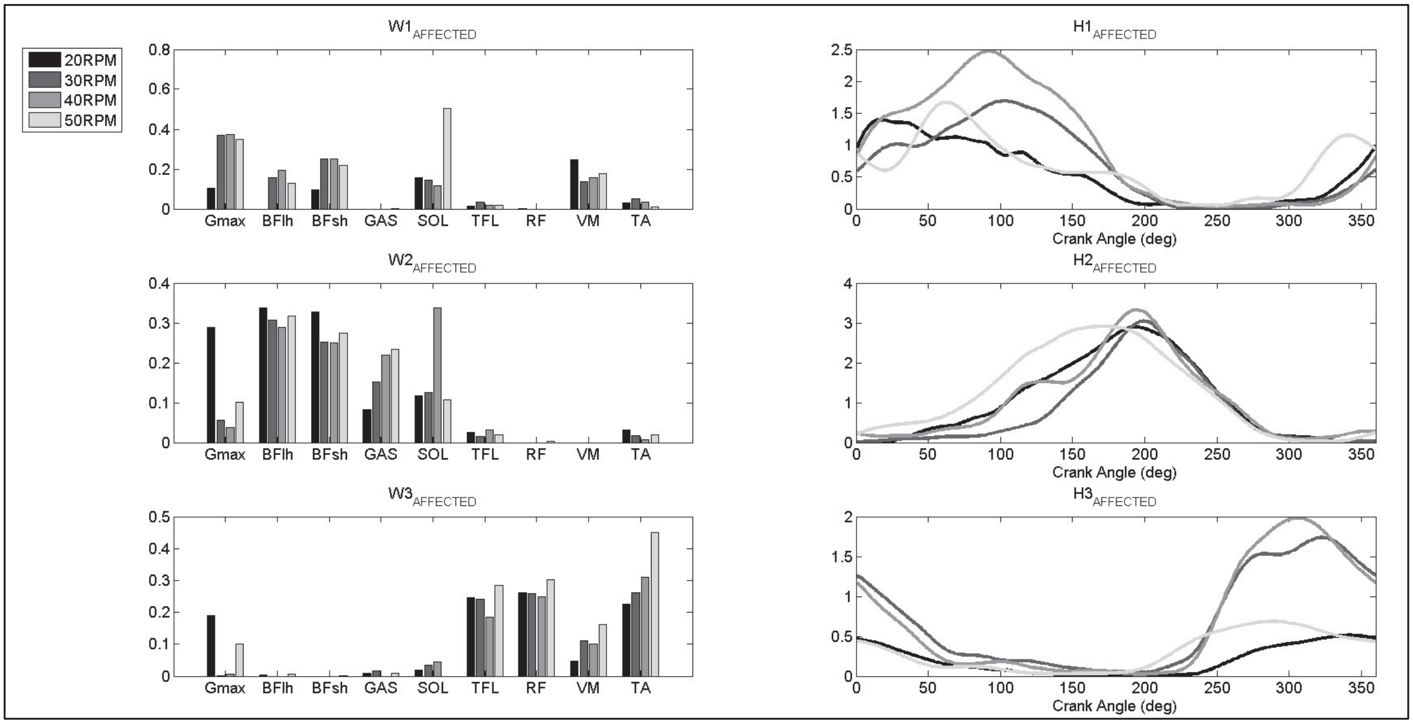

PATIENT P8

Group: C – 4 modules

Age (years): 72

Days post Stroke: 19

Type of Stroke: ischemic

Motricity Index (leg subscale): 75

Gait Speed: 0.39 m/s

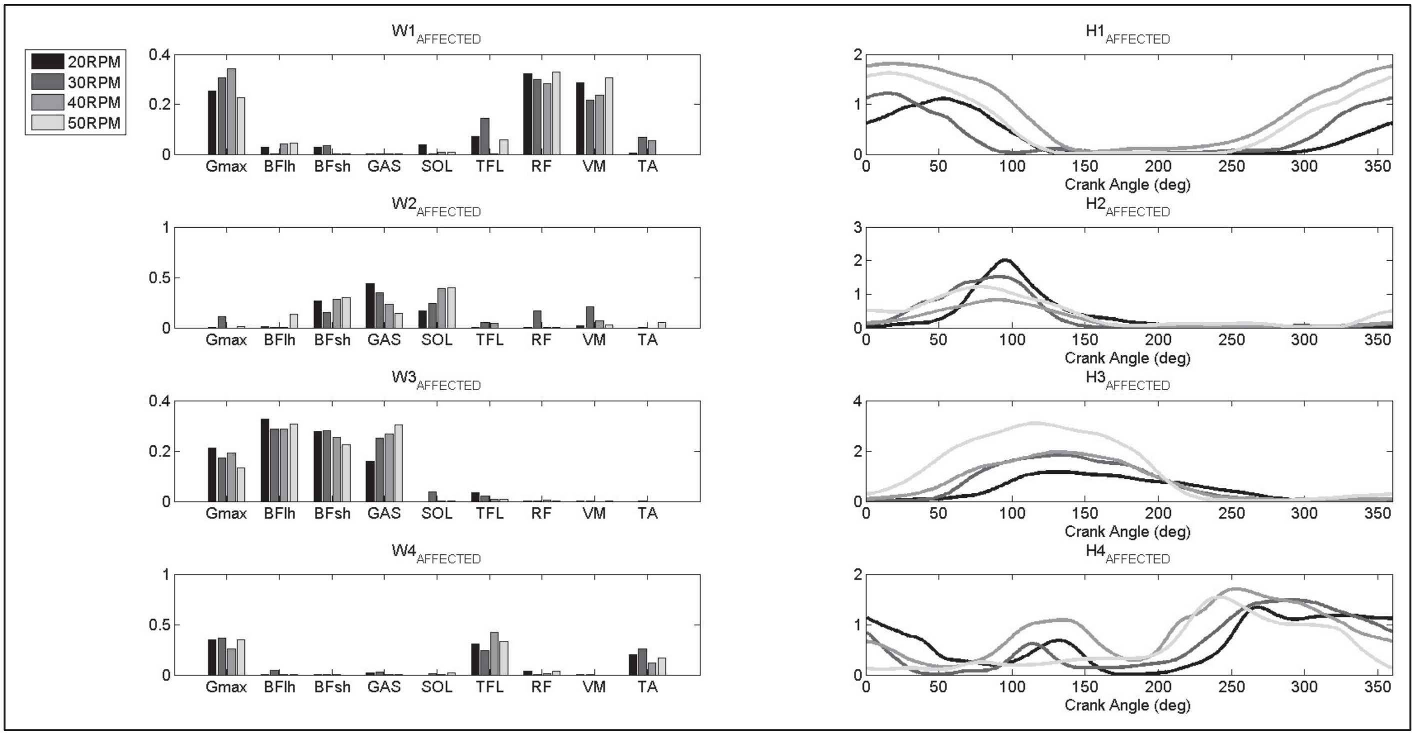

PATIENT P9

Group: B – 3 modules

Age (years): 74

Days post Stroke: 17

Type of Stroke: ischemic

Motricity Index (leg subscale): 75

Gait Speed: 1.18 m/s

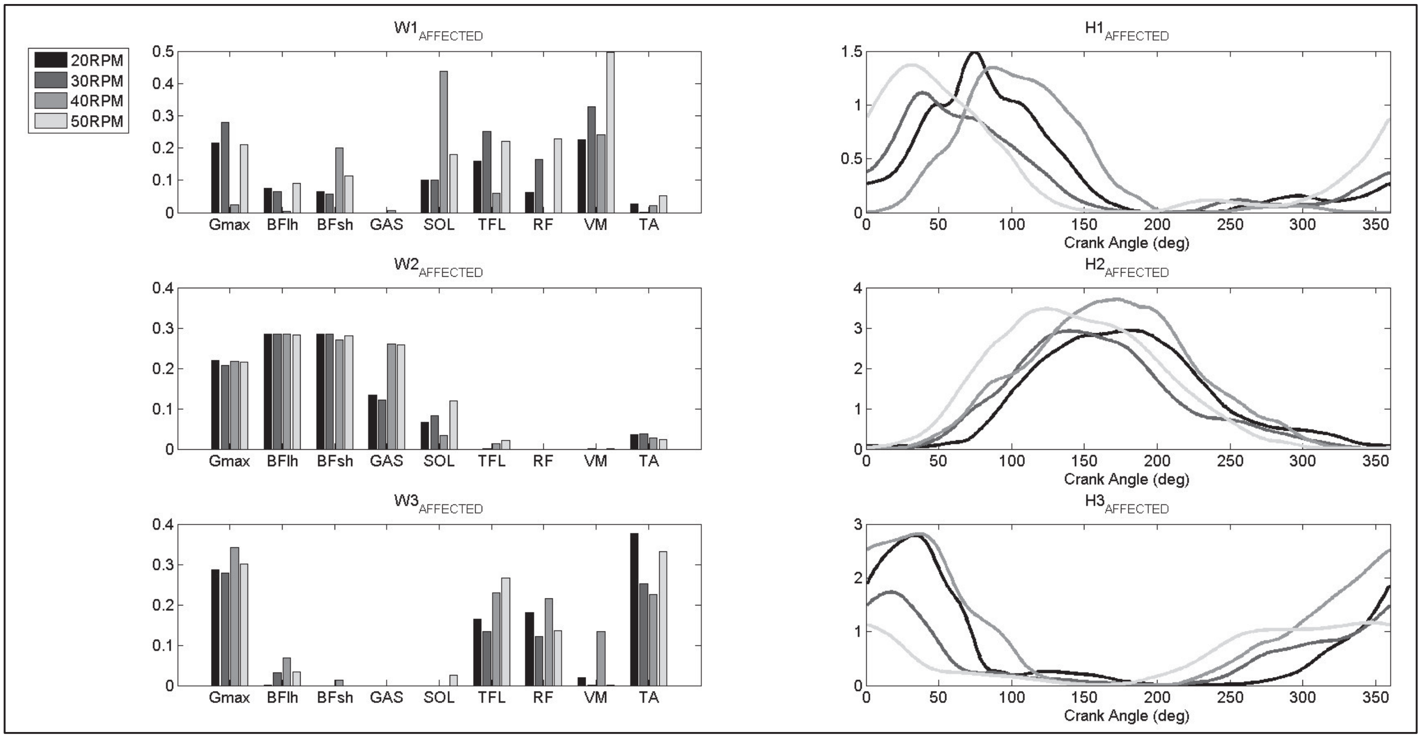

PATIENT P10

Group: A – 2 modules

Age (years): 47

Days post Stroke: 9

Type of Stroke: ischemic

Motricity Index (leg subscale): 52

Gait Speed: 0.44 m/s

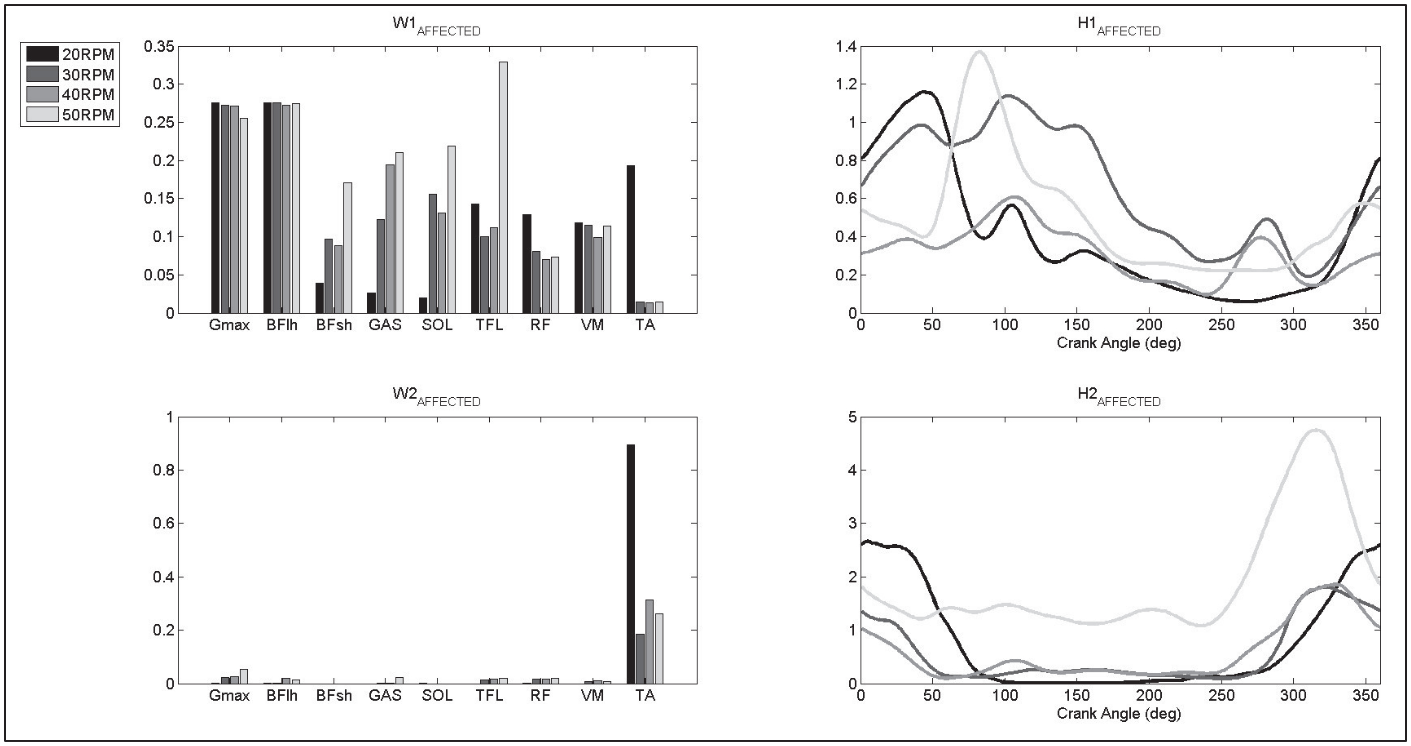

PATIENT P11

Group: C – 4 modules

Age (years): 73

Days post Stroke: 18

Type of Stroke: ischemic

Motricity Index (leg subscale): 75

Gait Speed: 0.79 m/s

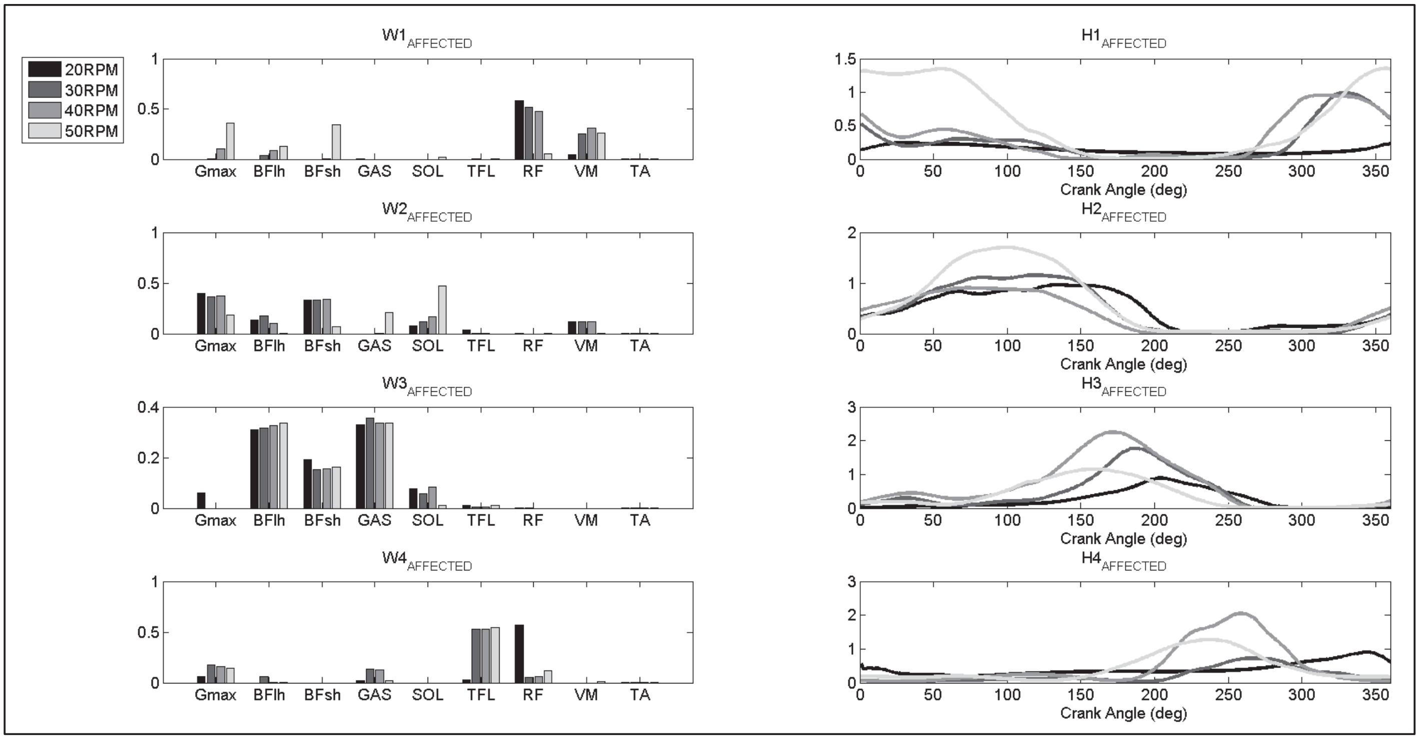

PATIENT P12

Group: B – 3 modules

Age (years): 82

Days post Stroke: 10

Type of Stroke: ischemic

Motricity Index (leg subscale): 75

Gait Speed: 0.48 m/s

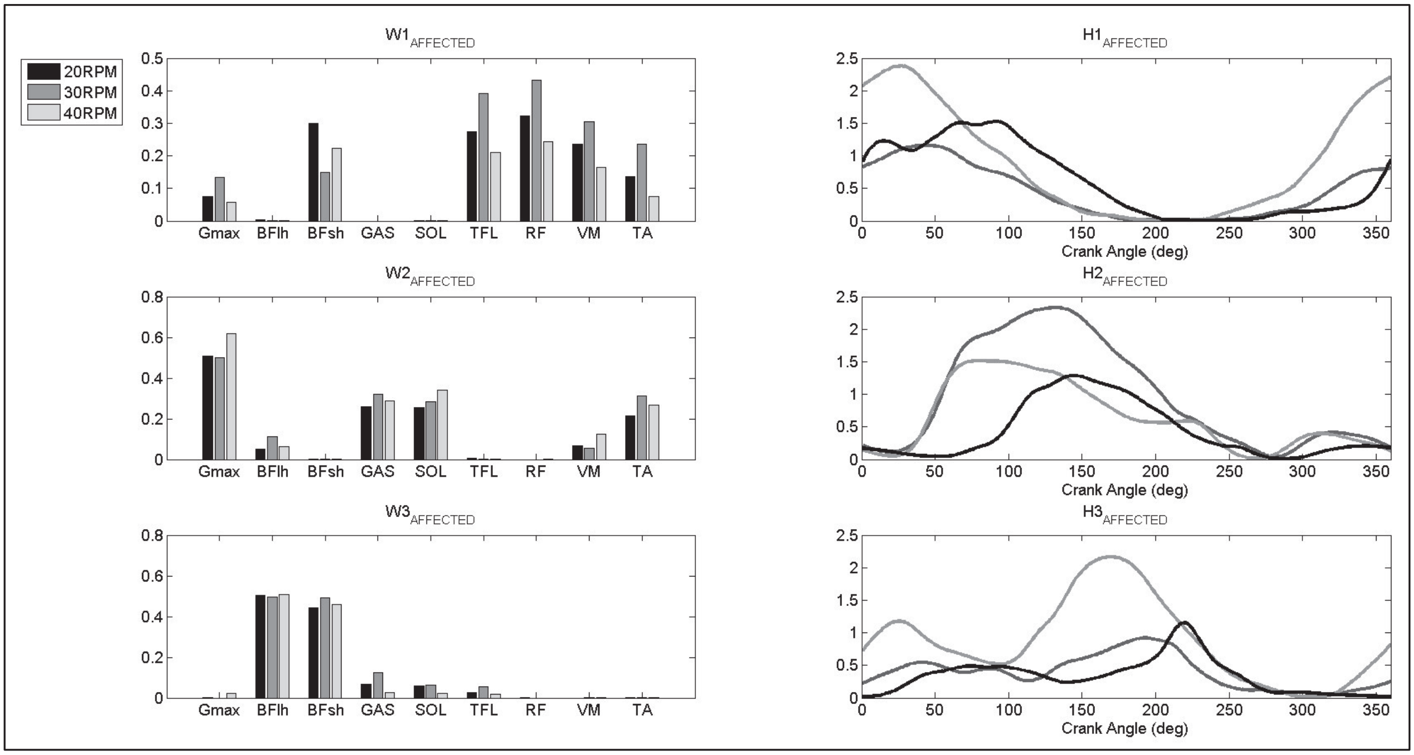

Missing trial at 50 RPM

PATIENT P13

Group: A – 2 modules

Age (years): 76

Days post Stroke: 12

Type of Stroke: hemorragic

Motricity Index (leg subscale): 75

Gait Speed: 0.77 m/s

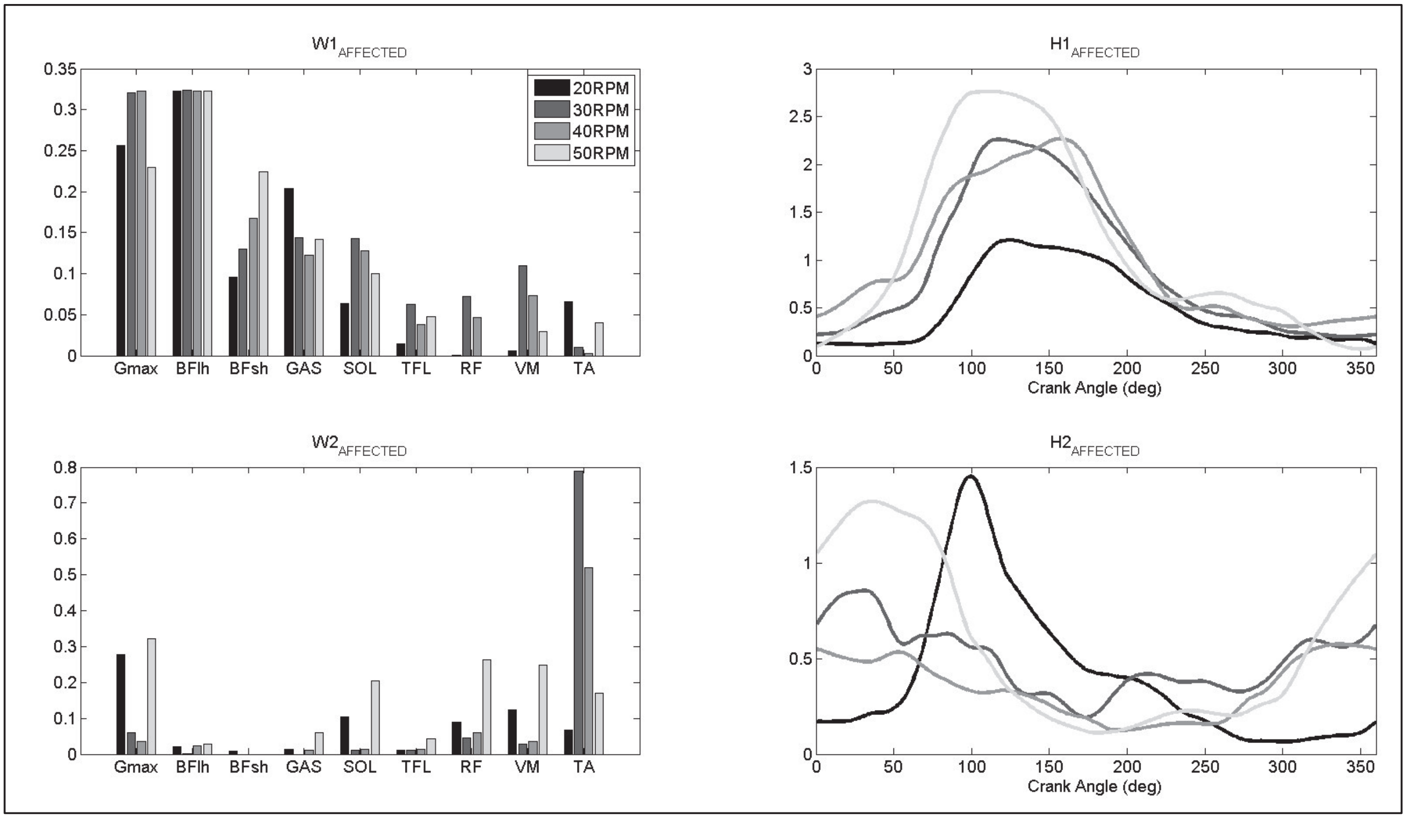

PATIENT P14

Group: C – 4 modules

Age (years): 58

Days post Stroke: 18

Type of Stroke: hemorragic

Motricity Index (leg subscale): 63

Gait Speed: 0.82 m/s

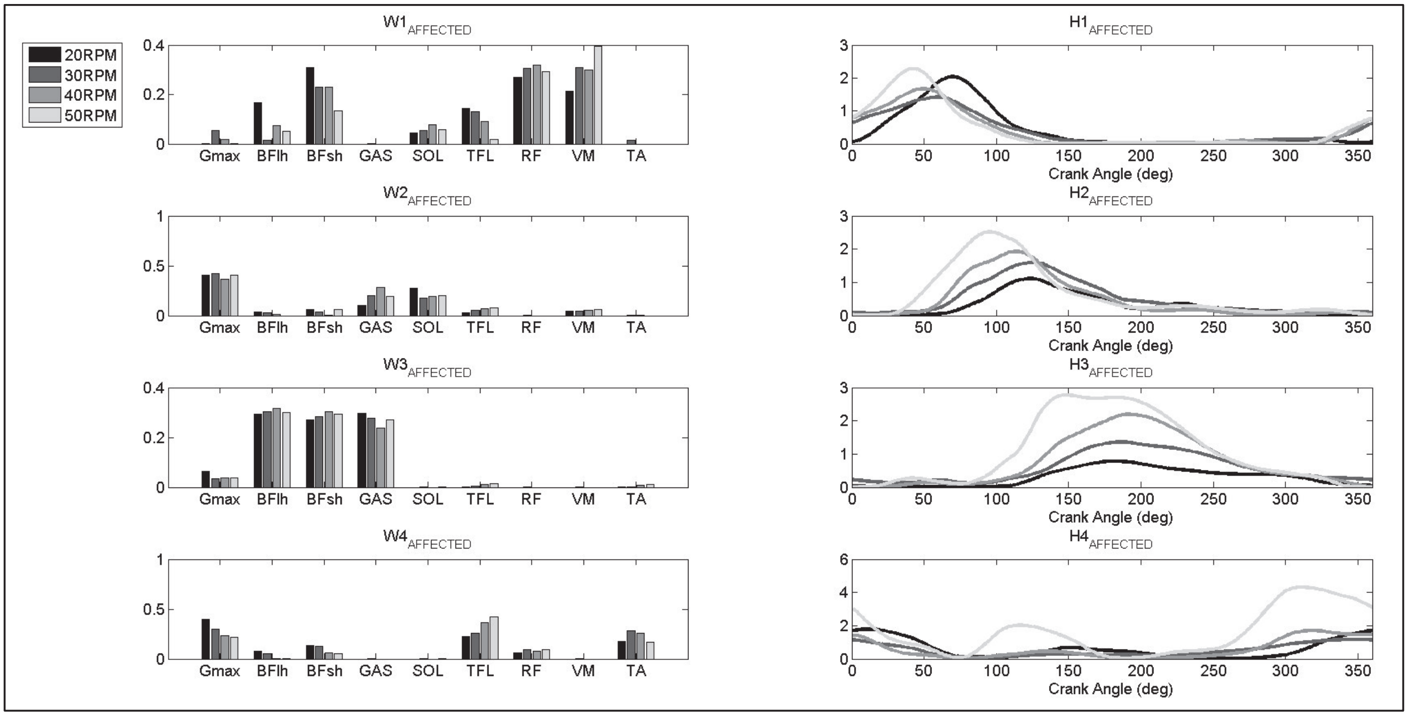

PATIENT P15

Group: B – 3 modules

Age (years): 84

Days post Stroke: 15

Type of Stroke: ischemic

Motricity Index (leg subscale): 63

Gait Speed: 0.63 m/s

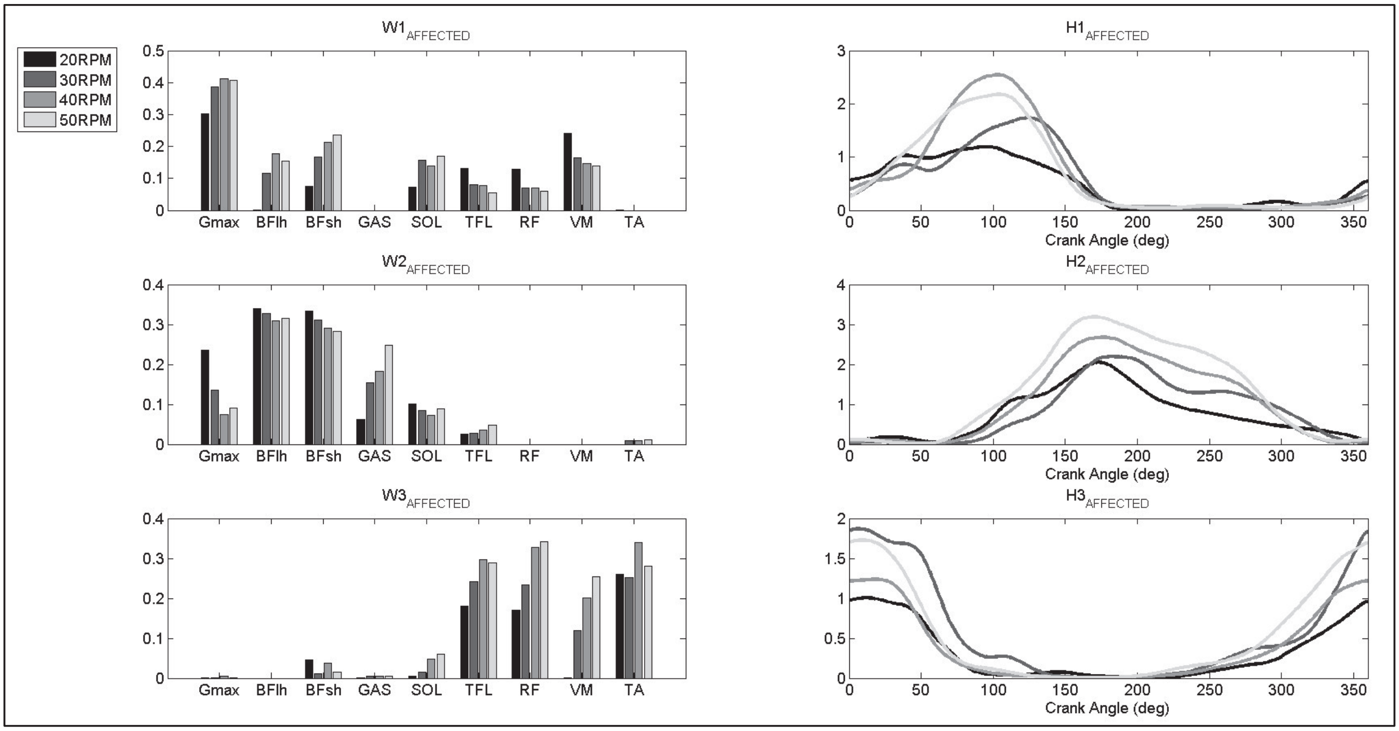

PATIENT P16

Group: B – 3 modules

Age (years): 65

Days post Stroke: 78

Type of Stroke: ischemic

Motricity Index (leg subscale): 69

Gait Speed: 0.52 m/s

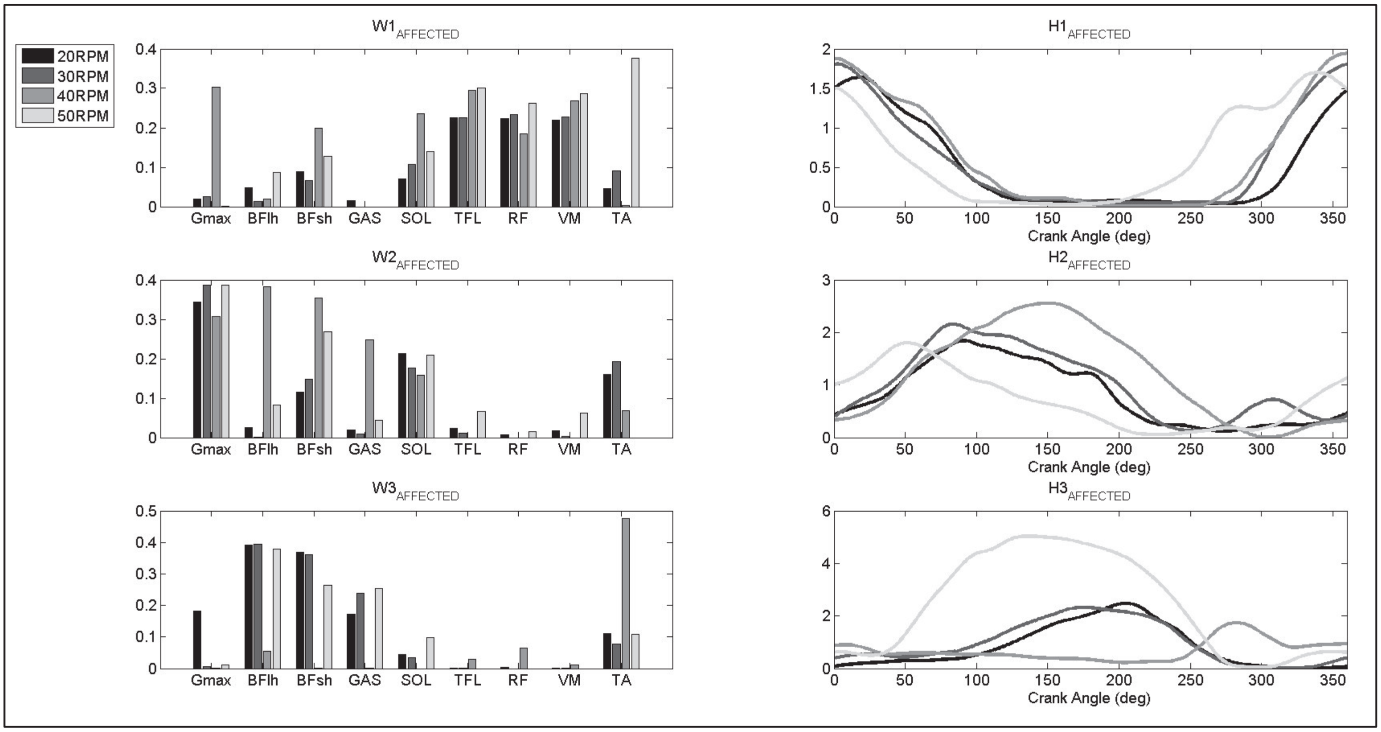

Table S1. Results of the mixed two-factor ANOVA carried out on the VR values. P-values achieved for each muscle and both effects (cadence: 20-50 RPM, and leg: dominant/non-dominant for healthy, affected/unaffected for patients) are reported.

	Gmax	BFlh	BFsh	GAS	SOL	TFL	RF	VM	TA
**Cadence effect**	0.012*	0.051	0.257	0.114	0.483	0.086	0.175	0.143	0.087
**Leg effect**	0.883	0.003†	0.031‡	0.559	0.104	0.120	0.144	0.371	0.416

* The post-hoc analysis revealed that 20 RPM is significantly different from 50 RPM, and 30 RPM from 40 RPM.

† The post-hoc analysis revealed that the healthy dominant and non-dominant leg are significantly different from the patients' unaffected leg.

‡ The post-hoc analysis did not reveal any significant paired differences.

Table S2. Results of the repeated-measures two-factor ANOVA applied to the synergy vectors extracted from the healthy subjects, when the number of synergies was fixed to 4. P-values of both cadence (20-50 RPM) and leg (dominant/non-dominant) effects are reported.

	Gmax	BFlh	BFsh	GAS	SOL	TFL	RF	VM	TA
**$W_{j,1}$ ***									
**Cadence effect**	0.375	0.540	0.566	0.303	0.094	0.794	0.048†	0.703	0.364
**Leg effect**	0.395	0.665	0.282	0.417	0.319	0.771	0.234	0.549	0.958
**$W_{j,2}$ ***									
**Cadence effect**	0.278	0.243	0.467	0.509	0.890	0.297	0.649	0.361	0.867
**Leg effect**	0.820	0.865	0.426	0.355	0.177	0.071	0.876	0.818	0.705
**$W_{j,3}$ ***									
**Cadence effect**	0.139	0.106	0.848	0.302	0.879	0.469	0.420	0.403	0.559
**Leg effect**	0.989	0.488	0.512	0.561	0.550	0.204	0.769	0.730	0.353
**$W_{j,4}$ ***									
**Cadence effect**	0.081	0.102	0.486	0.291	0.485	0.076	0.287	0.312	<0.001‡
**Leg effect**	0.141	0.382	0.603	0.913	0.265	0.669	0.749	0.784	0.630

* $W_{j,1}$ - $W_{j,4}$ represent the synergy vectors, with the index *j* representing the muscle.

† The post-hoc analysis revealed that 20 RPM is significantly different from 50 RPM.

‡ The post-hoc analysis revealed that 30 RPM is significantly different from 40 and 50 RPM.
